# Altered autophagic flux enhances inflammatory responses during inflammation-induced preterm labor

**DOI:** 10.1038/srep09410

**Published:** 2015-03-23

**Authors:** Varkha Agrawal, Mukesh K. Jaiswal, Timothy Mallers, Gajendra K. Katara, Alice Gilman-Sachs, Kenneth D. Beaman, Emmet Hirsch

**Affiliations:** 1 Department of Obstetrics and Gynecology, NorthShore University HealthSystem, Evanston, IL; 2 Department of Microbiology and Immunology, Rosalind Franklin University of Medicine and Science, North Chicago, IL, USA; 3 Department of Obstetrics and Gynecology, Pritzker School of Medicine, University of Chicago, Chicago, IL

## Abstract

Cellular organelles and proteins are degraded and recycled through autophagy, a process during which vesicles known as autophagosomes fuse with lysosomes. Altered autophagy occurs in various diseases, but its role in preterm labor (PTL) is unknown. We investigated the role of autophagic flux in two mouse models of PTL compared to controls: 1) inflammation-induced PTL (IPTL), induced by toll-like receptor agonists; and 2) non-inflammation (hormonally)-induced PTL (NIPTL). We demonstrate that the autophagy related genes Atg4c and Atg7 (involved in the lipidation of microtubule-associated protein 1 light chain 3 (LC3) B-I to the autophagosome-associated form, LC3B-II) decrease significantly in uterus and placenta during IPTL but not NIPTL. Autophagic flux is altered in IPTL, as shown by the accumulation of LC3B paralogues and diminishment of lysosome associated membrane protein (LAMP)-1, LAMP-2 and the a2 isoform of V-ATPase (a2V, an enzyme involved in lysosome acidification). These alterations in autophagy are associated with increased activation of NF-κB and proinflammatory cytokines/chemokines in both uterus and placenta. Similar changes are seen in macrophages exposed to TLR ligands and are enhanced with blockade of a2V. These novel findings represent the first evidence of an association between altered autophagic flux and hyper-inflammation and labor in IPTL.

In eukaryotes cellular organelles and proteins undergo turnover and recycling through a pathway known as autophagy^1^. Organelles and proteins marked for degradation are sequestered into autophagosomes (double-layer membrane-limited vesicles), which then fuse with lysosomes to form autophagolysosomes (which have a single membrane layer) in which the contents are degraded. During this process biomolecules such as amino acids, free fatty acids and nucleotides are recycled for macromolecule biosynthesis and ATP production. A minimal essential level of autophagy is required for organelle turnover and management of cell growth and energy production^2^,^3^. This autophagic flux is modulated by various cellular stresses, including nutrient deprivation, reactive oxygen species, hypoxia, protein aggregates, DNA and organelle damage, or intracellular pathogens^3^.

Human diseases associated with defective autophagy and autophagy related genes include Parkinson's disease, Huntington's disease, Alzheimer's disease, aging, diabetes, myopathies, muscle atrophy, inflammatory bowel disease, obesity, cystic fibrosis, sepsis and cancer^4^,^5^,^6^. Women with preeclampsia and intrauterine growth restriction (IUGR) have higher placental levels of LC3B and other proteins related to autophagy in comparison to normal pregnant women^7^,^8^.

Various components of the autophagy machinery have positive and negative interactions with the innate immune system. Toll-like receptors (TLRs) are the first subclass of pattern recognition receptors (PRRs, which recognize pathogenic molecular motifs and activate innate immunity) demonstrated to be connected with autophagy^6^,^9^. Autophagy is triggered as a defense mechanism for eliminating intracellular pathogens after engagement of TLRs by their ligands^10^,^11^,^12^. TLRs involved in autophagy have been identified in various cell types (macrophages, dendritic cells, and neutrophils) including TLR2/TLR1 heterodimer, TLR3, TLR4 TLR7/8, and TLR9. Lipopolysaccharide (LPS, a TLR4 ligand) and polyinosinic:cytidylic acid (poly(I:C), a TLR3 ligand) modulate autophagy via enhancing autophagosome formation in monocytes and macrophages^10^,^11^,^12^ while triggering activation of the master inflammatory regulator nuclear factor (NF)-κB and MAP kinase. NF-κB activation is itself a negative regulator of autophagy^13^, whereas different MAP kinases have differential effects on autophagy. The activation of p38 and extracellular signal-regulated kinase (ERK) disturb the maturation phase of autophagy, playing a negative- and a positive- role, respectively^14^,^15^. IκB kinase (IKK), a kinase upstream of NF-κB is an inducer of NF-κB activation and is directly regulated by autophagy^16^,^17^.

Preterm birth is one of the most significant causes of neonatal mortality and morbidity. About 40% of cases of preterm labor (PTL) are associated with infection within the gestational compartment^18^,^19^. We and others have shown that PTL can be induced in animal models by pathogen-derived TLR ligands for TLR4 (LPS^20^) TLR2 (peptidoglycan, PGN^21^), TLR3 (poly(I:C))^21^, and in a synergistic manner, TLR2+TLR3^20^,^22^,^23^,^24^.

Vacuolar ATPase (V-ATPase) is an enzyme situated in the lysosomal membrane^25^ and plasma membranes of specific cells^26^,^27^ and reproductive tissues^26^,^28^,^29^,^30^,^31^,^32^. The key function of V-ATPases is the acidification of intracellular compartments such as lysosomes, a process vital for various cellular functions, including autophagosome turnover^26^. Inhibitors of V-ATPase, such as bafilomycin A1, hinder autophagosome maturation by inhibiting fusion with lysosomes^33^. The a2 isoform of the ‘a’ subunit of vacuolar ATPase is known as a2V-ATPase (a2V)^26^ and has been shown to play a role in inflammation-induced preterm labor^32^.

In the present study, we sought to determine the role of autophagy in preterm labor. We examined the expression of molecules related to autophagy in inflammation-induced preterm labor (IPTL) and non-inflammation (hormonally) induced preterm labor (NIPTL) in the mouse. We also investigated the role of a2V in autophagy regulation during preterm labor. Our findings provide novel information showing altered autophagic flux during IPTL. The evidence suggests that this alteration occurs through suppression of a2V and lysosomal fusion and leads to enhanced inflammation and possibly is linked to labor.

## Results

### Autophagy-related proteins are decreased and lead to altered autophagy in IPTL

Microtubule-associated protein 1 light chain 3 (LC3) B, specifically the lipidated form (LC3B-II), is a widely used marker to monitor autophagy and correlates with the number of autophagosomes. For the formation of LC3B-II bearing autophagosomes in the cytoplasm an ensemble of autophagy-related (Atg) proteins (Atg3, Atg4 and Atg7) is required^3^,^34^. Therefore, we tested the level of Atg3, Atg4c and Atg7 in IPTL and NIPTL. IPTL was induced using two different intrauterine TLR ligand regimens: LPS^20^,^35^ or the combination of PGN+poly(I:C)^20^,^24^. NIPTL was induced using the progesterone receptor antagonist mifepristone subcutaneously. Either of these interventions leads to delivery within 24 hours or less^36^,^37^. Placentas and uterine tissues (from regions inclusive of the decidual caps underlying placental attachment sites) were harvested 8–10 hours after surgery.

**Figure 1:**
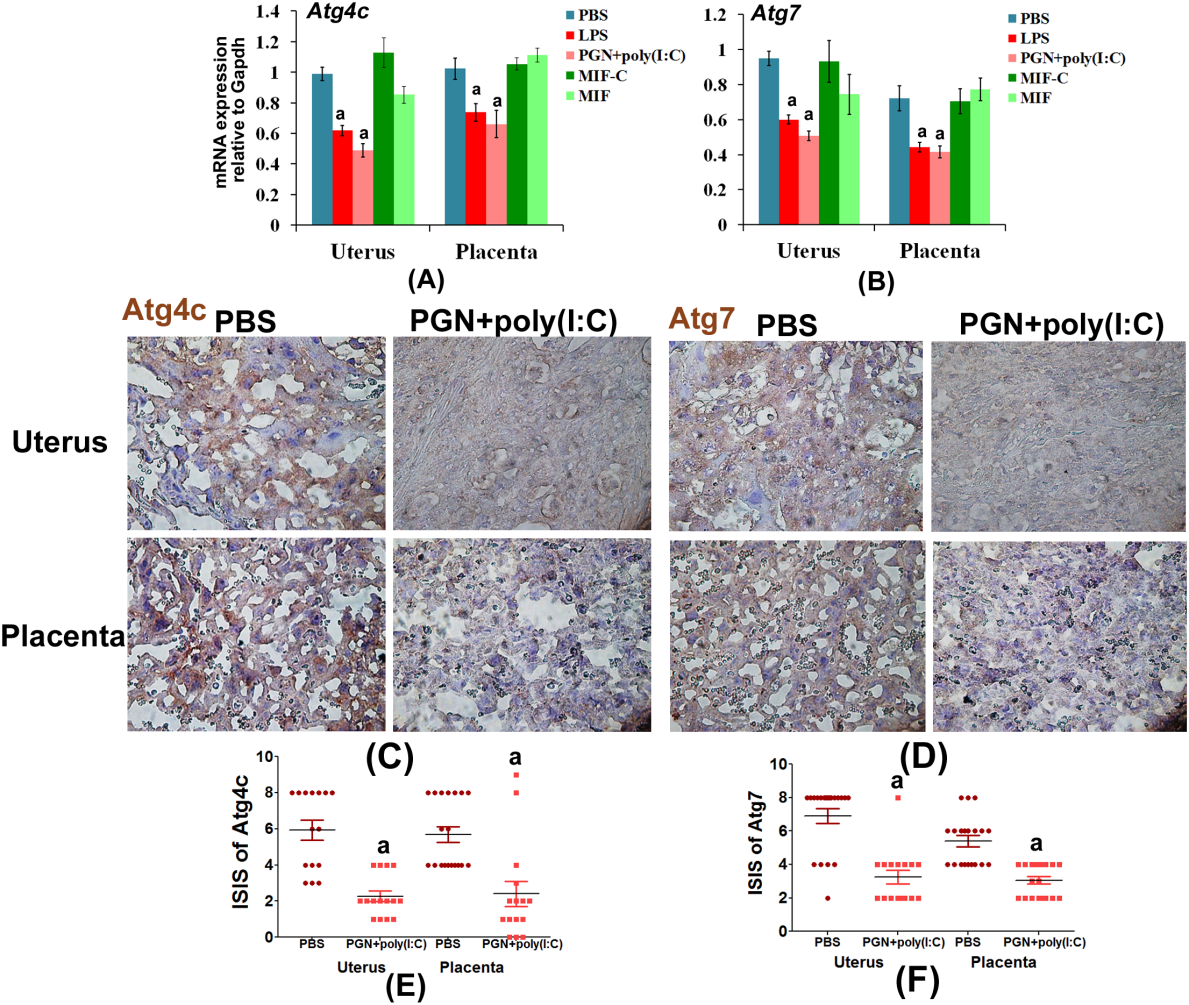
Autophagy-related proteins in various forms of labor. Panels A and B show mRNA expression of Atg4c and Atg7 in uterus and placenta recovered from preterm labor groups and controls. N = 6–11 each group. Panels C and D show tissue distribution of Atg4c and Atg7 protein, respectively. Corresponding immunostaining index scores are shown in E and F [(ISIS) = Stained area score (SAS) X Immunostaining intensity score (IIS)]. Six tissue sections per animal were analyzed. Original magnification: 400X. PBS, LPS and PGN+poly(I:C): intrauterine injections on day 14.5; MIF-C: subcutaneous DMSO control, MIF: subcutaneous mifepristone in DMSO on day 14.5. Error bars = ±SEM. ^a^P ≤ 0.05 Significant difference vs. respective control.

A significant decrease of Atg4c and Atg7 mRNA occurs in LPS-treated and PGN+poly(I:C)-treated uterus and placenta in comparison to control tissues (Fig. 1A and B). Expression remained unchanged in the NIPTL group in comparison to control. Immunohistochemical analysis also confirmed that Atg4c and Atg7 protein levels are decreased in PGN+poly(I:C)-treated (Fig. 1C–F) and LPS-treated (see Supplementary Fig. S1 online) uterus and placenta compared to their controls. Atg3 levels were unchanged as shown by western blot and immunohistochemistry (see Supplementary Fig. S2 online). Isotype controls are shown.

**Figure 2:**
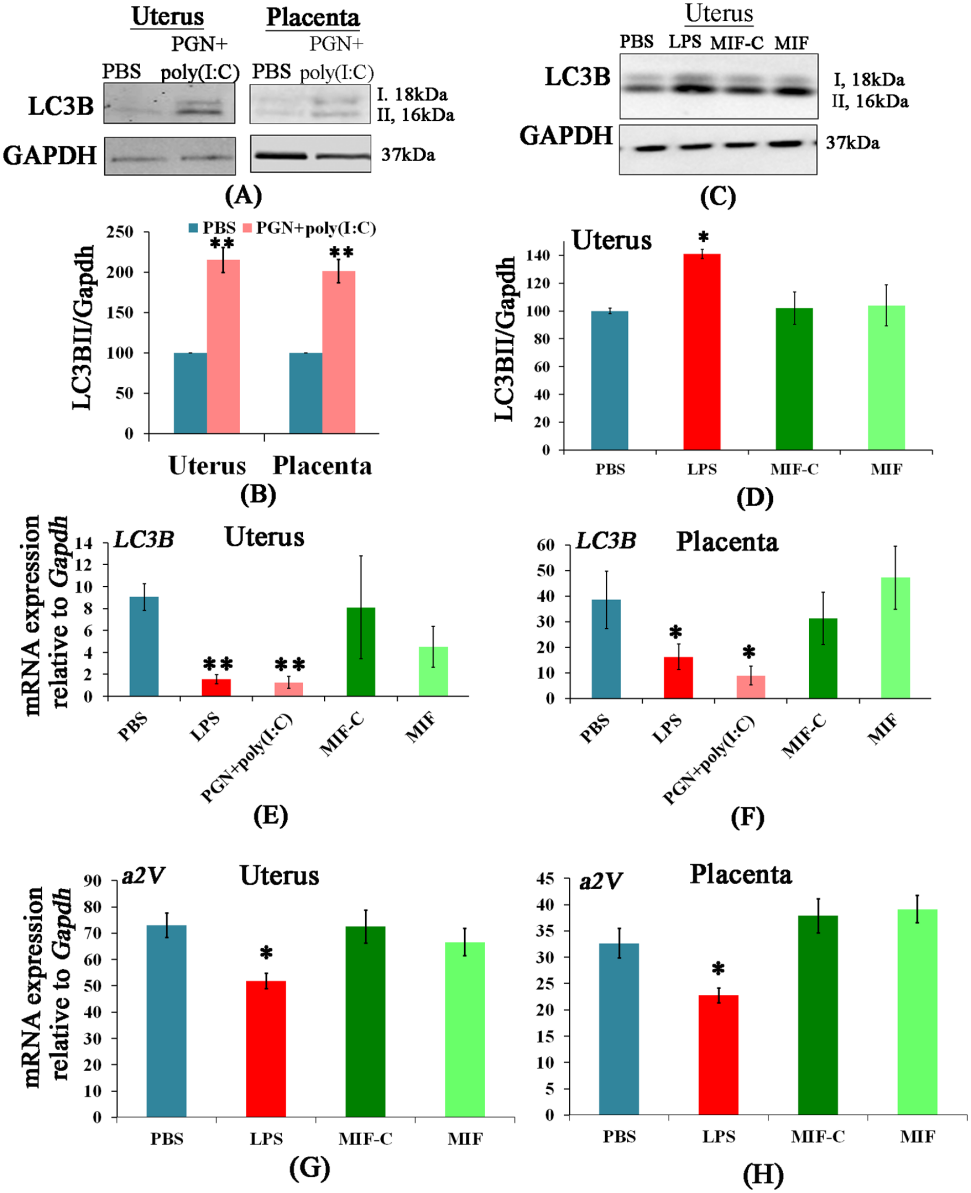
LC3B protein is increased and a2V mRNA is decreased in inflammation-induced preterm labor. Panels A and C show western blots of LC3B-I, LC3B-II and GAPDH in uterus and placenta recovered from preterm labor groups and controls with corresponding densitometric analysis (LC3B-II/GAPDH) in panels B and D. LC3B-I densitometric analysis shows a similar pattern (data not shown). N = 4–5 each group. Panels E and F show LC3B and panels G and H show a2V mRNA expression in uterus and placenta recovered from preterm labor groups and controls. N = 6–11 each group. PBS, LPS and PGN+poly(I:C): intrauterine injections on day 14.5; MIF-C: subcutaneous DMSO control, MIF: subcutaneous mifepristone in DMSO on day 14.5. Error bars = ±SEM. *P ≤ 0.05, **P ≤ 0.01 Significant difference vs. respective control. Full-length blots are presented in Supplementary Figure 12.

Given that the levels of Atg4c and Atg7 (i.e. proteins which, through lipidation of LC3B, play a role in formation of autophagosomes) are decreased in the LPS-treated and PGN+poly(I:C)-treated uterus and placenta, we next evaluated the level of the autophagosome marker LC3B in PTL groups. Using an antibody that detects the LC3B paralogues (LC3B-I and -II), western blot analysis was carried out in uterus and placenta. Compared with control, LC3B-I and -II protein were significantly increased in PGN+poly(I:C) and LPS-treated uterus and in PGN+poly(I:C)-treated placenta (Fig. 2A–D), while LC3B mRNA expression was significantly decreased (Fig. 2E and F), suggesting that increased accumulation of LC3B paralogues is not due to the increased transcription. There were no changes in the NIPTL group in comparison to its control (Fig. 2C–F).

Thus, in IPTL the increase of LC3B-I and -II proteins points toward a possible alteration in autophagic flux. Specifically, the increase of LC3B-I level suggests that the process of conversion of LC3B-I to LC3B-II by lipidation is hindered, in part because of decreased production of Atg4c and Atg7. Despite this impairment in LC3B-I lipidation, there is an increase in LC3B-II (a marker for mature autophagosomes), which might be due to either increased autophagosome production or impaired autophagosome turnover. Importantly, monitoring only autophagosome number is not always a clear indicator of autophagic flux, as this might be influenced by either altered generation of autophagosomes and/or changes in autophagosome maturation and turnover^38^. Therefore, we next evaluated lysosomes and expression and function of the lysosomal enzyme vacuolar ATPase, which is essential for normal autophagosome turnover.

### Decreased a2V expression and increased LC3B in IPTL leads to altered autophagic flux

The lysosomal enzyme vacuolar ATPase (V-ATPase) is responsible for maintenance of an acidic state in intracellular compartments (such as lysosomes, endosomes and secretory vesicles) and resides on the plasma membrane of certain specialized cells (such as macrophages). a2V (the a2 isoform of V-ATPase) is necessary for various cellular functions such as targeting of lysosomal enzymes, receptor-mediated endocytosis, and protein processing and degradation^39^. To test whether a2V plays a role in the regulation of autophagy in IPTL and NIPTL, expression of a2V was determined.

**Figure 3:**
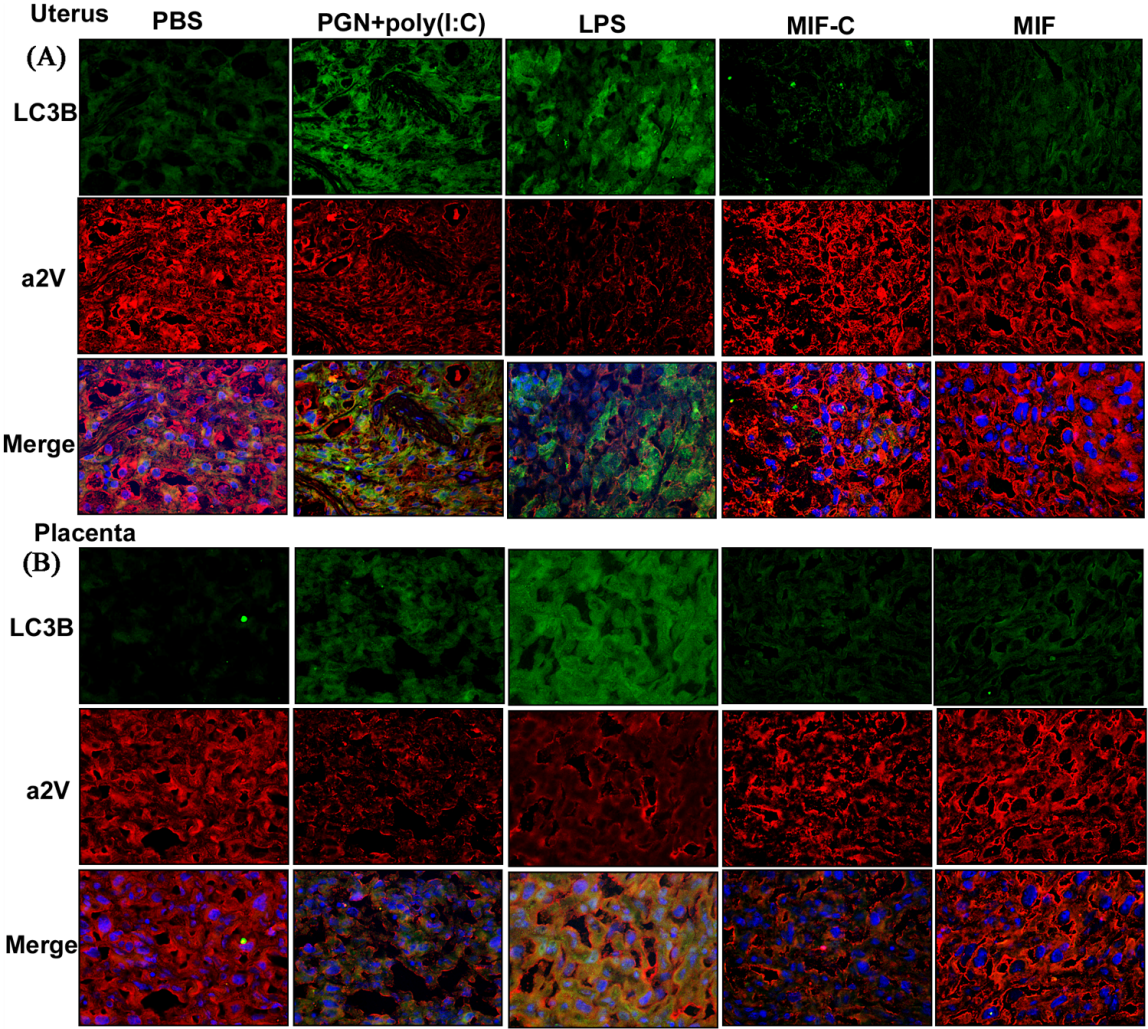
Expression of a2V is decreased and LC3B is increased in inflammation induced preterm labor. Uterus shown in (A) and placenta in (B). LC3B stained in green; a2V stained in red. Nuclei stained with DAPI (blue) in merged images. N = 4–5 each group. Six sections per animal were analyzed. Original magnification: 200X. PBS, LPS and PGN+poly(I:C): intrauterine injections on day 14.5; MIF-C: subcutaneous DMSO control on day 14.5; MIF: subcutaneous mifepristone in DMSO on day 14.5.

The mRNA expression of a2V was decreased significantly in IPTL induced by PGN+poly(I:C) (reported in Ref. 32) and LPS (Fig. 2G and H) in uterus and placenta in comparison to control. There was no change in the NIPTL group in comparison to control (Fig. 2G and H). The above patterns of expression (increased LC3B and decreased a2V) were confirmed on the protein level by immunofluorescence. Double immunofluorescence staining shows that in decidual and placental tissues LC3B is increased and a2V is decreased in both LPS-induced and PGN+poly(I:C)-induced IPTL (Fig. 3). Additionally, higher magnification images show the accumulation of LC3B in a punctal pattern characteristic of autophagosomes (see Supplementary Fig. S3 online). There was no change in the NIPTL group compared to its control. Thus, the decrease of a2V is linked with a higher number of autophagosomes (LC3B), which supports the existence of a functional role for a2V in the regulation of autophagic flux during IPTL.

Macrophages are considered a critical cell type responsible for labor, and they infiltrate gestational tissues in IPTL^32^,^40^,^41^. Therefore, we characterized the involvement of the above autophagy pathways in macrophages. Double immunofluorescence staining of the macrophage marker F4/80 and LC3B demonstrates that the decidua is highly infiltrated with macrophages in IPTL, and that these macrophages express high levels of LC3B (see Supplementary Fig. S4 online). Isotype controls are also shown.

To confirm the function of autophagy pathways and their dependence on a2V in macrophages, the RAW 264.7 mouse macrophage cell line was treated with a mouse monoclonal anti-a2V antibody. Blocking of a2V leads to decreased levels of Atg4c and Atg7 mRNA, as does exposure to either LPS or PGN+poly(I:C) (see Supplementary Fig. S5 online). Blocking of a2V increased the level of LC3B, whether in the absence or presence of PGN+poly(I:C) or LPS (see Supplementary Fig. S6 online). Isotype control is also shown (see Supplementary Fig. S7 online). Bafilomycin A1, an inhibitor of V-ATPase which blocks the fusion of autophagosomes to lysosomes and prevents the complete turnover of autophagosomes, gives a response similar to blocking with anti-a2V antibody (see Supplementary Fig. S6 online). These results support the hypothesis that a2V regulates autophagy by modulating the complete turnover of autophagosomes.

### Altered lysosomal function in IPTL, NIPTL and macrophages *in vitro*

**Figure 4:**
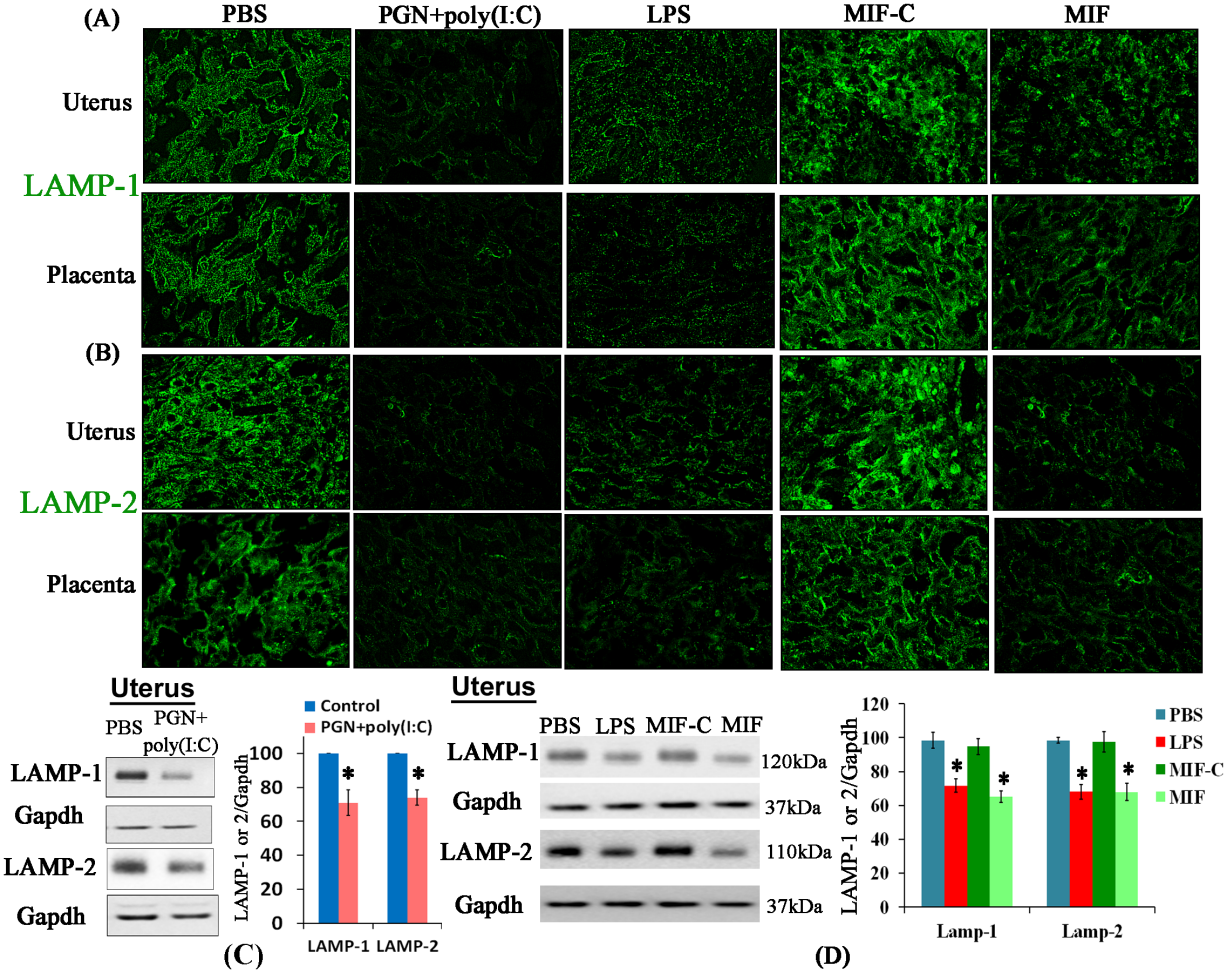
Expression of LAMP-1 and LAMP-2 is decreased in both inflammation-induced and non-inflammation-induced preterm labor. (A) LAMP-1 and (B) LAMP-2 (green) by immunofluorescence staining in uterus and placenta recovered from preterm labor and control groups. Six sections per animal were analyzed. Original magnification: 400X. Panels C and D show sample western blot and corresponding densitometric analysis of LAMP-1, LAMP-2 and GAPDH in uterus recovered from preterm labor and control groups. N = 4–5 each group. Error bars = ±SEM. *P ≤ 0.05 Significant difference vs. respective control. Full-length blots are presented in Supplementary Figure 12.

**Figure 5:**
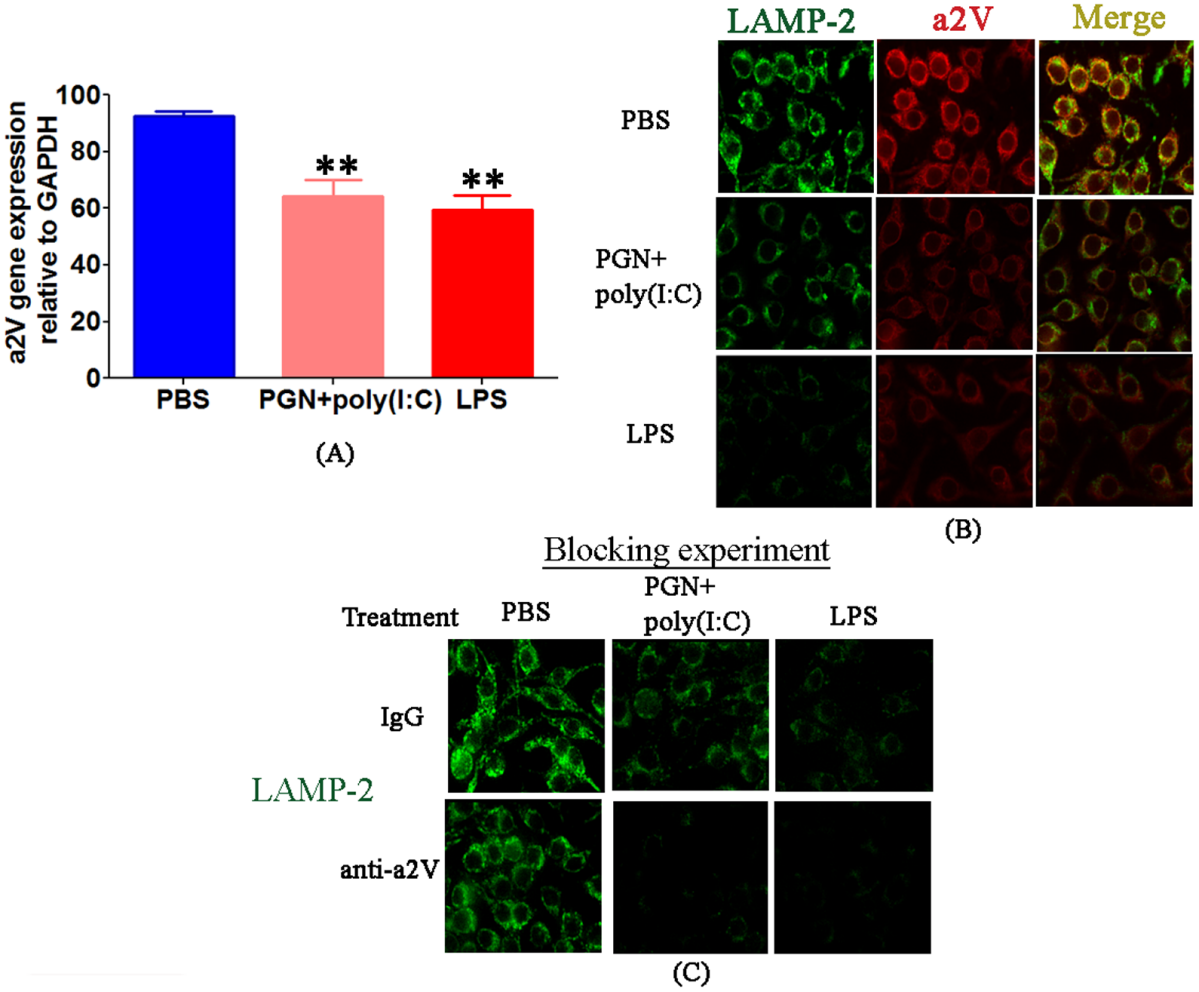
Decrease of a2V is associated with reduction of LAMP-2 in the RAW 264.7 mouse macrophage cell line. RAW 264.7 cells were treated for 30 min with PBS, PGN+poly(I:C) or LPS, followed by up to 2 h incubation with IgG control or anti-a2V antibody. (A) mRNA expression of a2V; (B) protein for LAMP-2 (green) and a2V (red); (C) LAMP-2 in the absence or presence of an a2V neutralizing antibody. Original magnification: 400X. Each experiment was done three times with triplicates. Error bars = ±SEM. **P ≤ 0.01 Significant difference vs. respective control.

To further elucidate the defect in autophagic flux in PTL, and to verify that these defects are associated with impairment of lysosomal fusion, we studied the distribution of the lysosomal markers lysosome associated membrane protein (LAMP)-1 and LAMP-2^42^ and the lysosomal enzyme a2V in uterus, placenta and macrophages. Both IPTL and NIPTL have significantly decreased levels of LAMP-1 and LAMP-2 protein in uterus and placenta compared with respective control tissues as observed by immunofluorescence and western blot (Fig. 4). Treatment of RAW 264.7 mouse macrophages with either PGN+poly(I:C) or LPS similarly diminishes the expression of both LAMP-2 and a2V (Fig. 5) within 2 h. Furthermore, blocking of a2V diminishes expression of LAMP-2 in both control conditions and with PGN+poly(I:C) or LPS treatment (Fig. 5). These findings were also confirmed *ex vivo* in decidual and placental cells extracted from day 14.5 pregnant mice (see Supplementary Fig. S8 online). Paralleling findings from the *in vitro* experiment, in uterine tissues from PGN+poly(I:C)-treated mice, a2V and LAMP-1 were co-localized with decreased expression in comparison to the control (see Supplementary Fig. S8 online).

Taken together, these results suggest that the altered autophagic flux observed in IPTL is due to disrupted formation of autolysosomes as well as decreased lysosomal function due to deficient a2V.

### Expression of NF-κB p65 is increased in IPTL

Autophagy maintains cellular homeostasis through blunting of inflammatory responses by regulating the degradation of NF-κB signaling components, resulting in the termination of NF-κB activation^43^. Thus, arrested autophagic flux would be expected to increase NK-κB activation, which has been shown previously to be central for multiple pro-labor pathways^44^.

**Figure 6:**
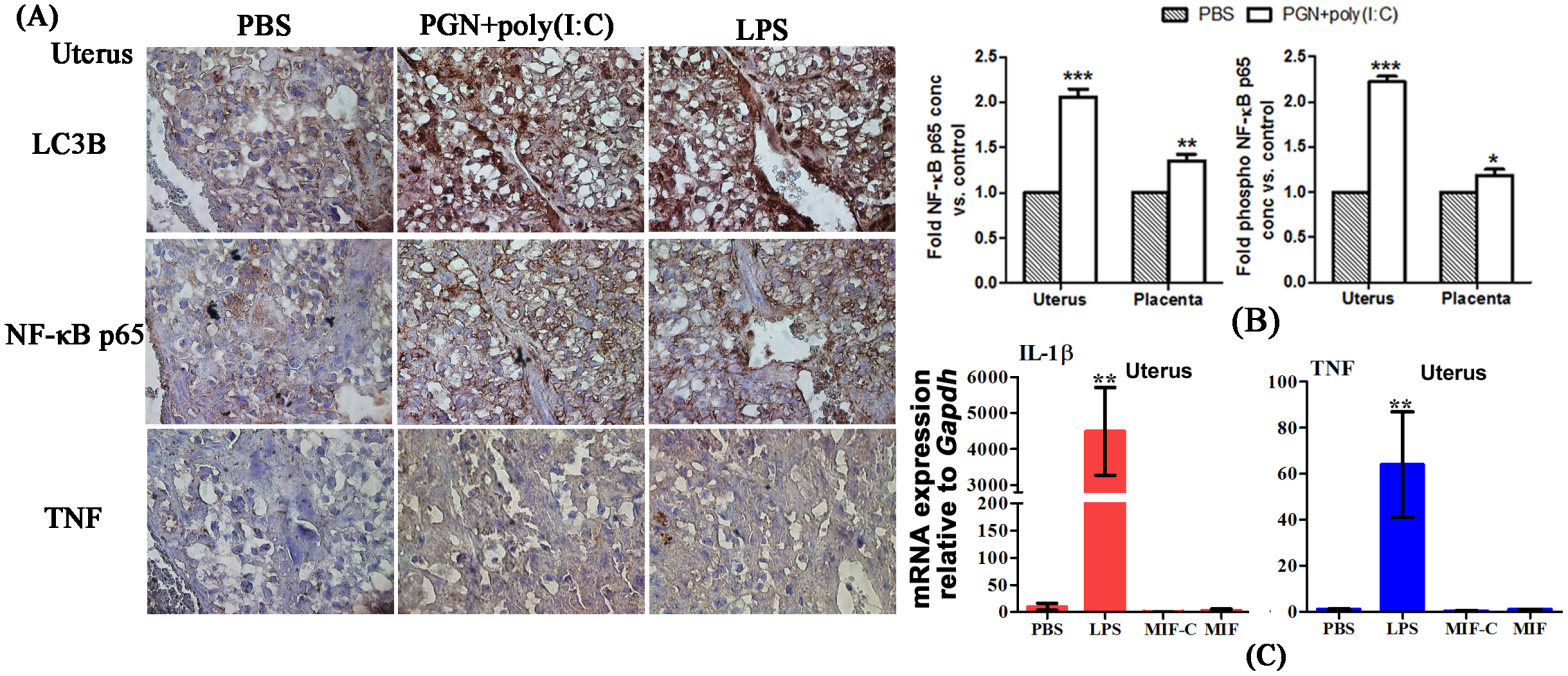
NF-κB p65 activation is increased in inflammation-induced preterm labor. (A) Distribution of LC3B, NF-κB p65 and TNF (brown) in serial sections of uterus recovered from PBS, PGN+poly(I:C) and LPS-treated animals. Six sections per animal were analyzed. Original magnification: 400X. (B) Total and phosphorylated NF-κB p65 by ELISA (control group set to 1). N = 4–5 each group. (C) mRNA expression of IL-1β and TNF in uterus by RT-PCR. N = 6–9 each group. PBS, LPS and PGN+poly(I:C): intrauterine injections on day 14.5; MIF-C: subcutaneous DMSO control, MIF: subcutaneous mifepristone in DMSO on day 14.5. Error bars = ±SEM. *P ≤ 0.05, **P ≤ 0.01, ***P ≤ 0.001 Significant difference vs. respective control.

To show the effect of altered autophagic flux in the context of inflammation, we tested the levels of LC3B, NF-κB p65 and TNF protein in uterus. Immunohistochemistry of serial sections of uterus showed increased LC3B, total NF-κB p65 and TNF in uterus from PGN+poly(I:C)-induced or LPS-induced PTL in comparison to control (Fig. 6A). ELISA confirms these increases in total and phosphorylated NF-κB p65 in uterus and placenta from animals treated with PGN+poly(I:C) (Fig. 6B). NF-κB p65 remained unchanged in the NIPTL group (data not shown). Similarly, the expression of IL-1β and TNF mRNA was increased in uterus from PGN+poly(I:C)-induced PTL [reported in Ref. 20,32] and LPS-induced PTL and remained unchanged in NIPTL (Fig. 6C).

### a2V blockade induces altered autophagic flux and enhances LPS- and PGN+poly(I:C)-induced NF-κB activation and expression of inflammatory markers

**Figure 7:**
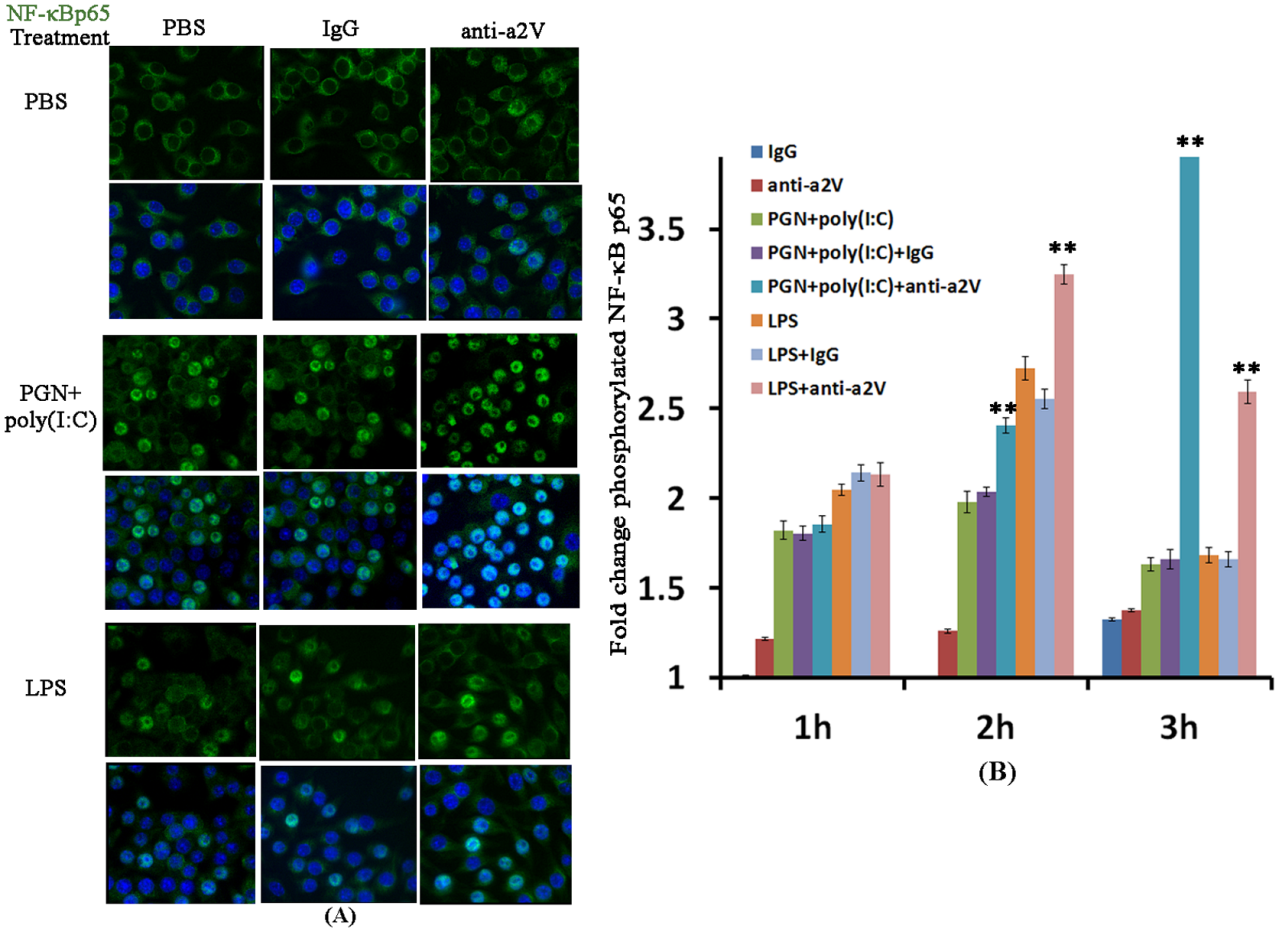
Blockade of a2V enhances baseline and LPS- and PGN+poly(I:C)-induced activation of NF-κB p65 in the RAW 264.7 mouse macrophage cell line. RAW 264.7 cells were treated for 30 min with PBS, PGN+poly(I:C) or LPS, followed by up to 3 h incubation with IgG control or anti-a2V antibody. Each experiment was done three times with triplicates. Shown is the translocation of NF-κB p65 (green) from the cytoplasmic to the nuclear compartment (blue) in 2 h (A). Original magnification: 400X. (B) Fold change in the concentration of phosphorylated NF-κB p65 by ELISA after 1 h, 2 h and 3 h of antibody incubation (IgG group set to 1). Error bars = ±SEM. **P ≤ 0.01 Significant difference between PGN+poly(I:C) or LPS treated with PBS/IgG vs. treated with anti-a2V.

We have previously reported that blocking of a2V by anti-a2V antibody enhances the expression of the inflammatory markers iNOS and TNF mRNA both at baseline and after treatment with PGN+poly(I:C) in RAW 264.7 macrophages^32^. Here we correlated this effect with NF-κB p65 activation and autophagy. RAW 264.7 mouse macrophages were treated with PBS, PGN+poly(I:C) or LPS for 30 min, followed by 1 h, 2 h or 3 h incubations with IgG control or anti-a2V antibody. As anticipated, PGN+poly(I:C) and LPS induce NF-κB p65 nuclear translocation, an effect that was amplified with blockade of a2V as measured by immunocytochemistry (Fig. 7). To confirm NF-κB p65 nuclear translocation, phosphorylated NF-κB p65 was also measured. As anticipated, PGN+poly(I:C) and LPS induce phosphorylation of NF-κB p65, an effect that was amplified and prolonged with blockade of a2V as measured by ELISA (Fig. 7).

A similar pattern of augmentation by a2V blockade of secretion into the conditioned medium of these cells over 5 h was seen for cytokines (TNF, IL-6, LIF, but not IL-2) and chemokines (G-CSF, GM-CSF, MCP1 and MIP-1α) by Luminex multiplex assay (see Supplementary Fig. S9 online). These findings were also confirmed *ex vivo* in decidual and placental cells extracted from day 14.5 pregnant mice (see Supplementary Fig. S10 online). Treatment with anti-a2V also enhanced LPS-induced mRNA expression of TNF and IL-1β in RAW 264.7 cells (see Supplementary Fig. S11 online).

These results support the sustained activation of NF-κB p65 during blockade of a2V in the presence of TLR ligands, supporting a role for altered autophagic flux in creating hyper-inflammation.

## Discussion

**Figure 8:**
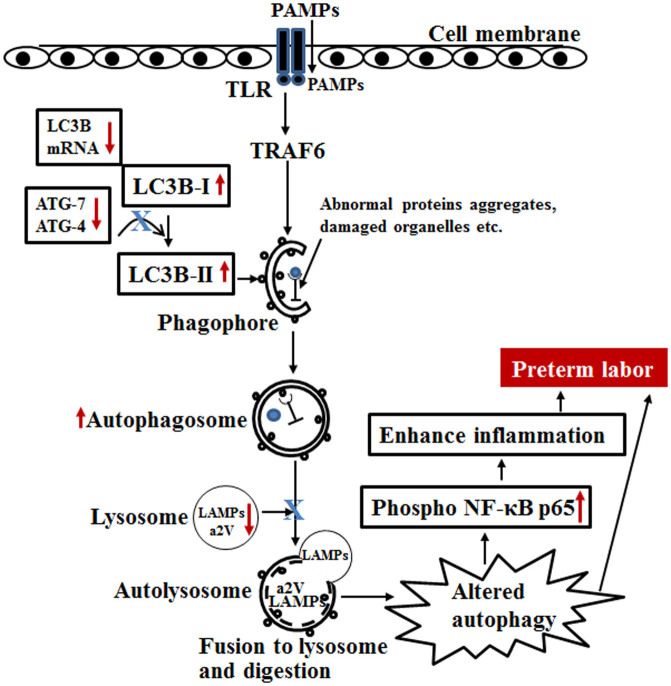
Model of autophagy in preterm labor. Red arrows represent observed changes; Blue X represents hypothesized mechanism based on our observations.

Autophagy is an intracellular process that maintains homeostasis by, among other processes, the removal of damaged organelles and proteins. The importance of autophagy in infectious diseases is emerging; however, its role in labor has not yet been elucidated. In the present study we report the following novel findings: (1) During inflammation-induced preterm labor (IPTL), autophagy in both uterus and placenta is altered, as reflected by diminished Atg enzymes and accumulation of LC3B paralogues; (2) The mechanism of this altered autophagy involves impairment of lysosomal fusion, as reflected by decreased levels of lysosome associated membrane protein (LAMP)-1 and LAMP-2, in part through diminished function of a2V; (3) Altered autophagy in both uterus and placenta and in the macrophages resident in these tissues may play a role in enhancing inflammatory processes in IPTL (Fig. 8).

Limited evidence suggests that baseline autophagy contributes to maintenance of healthy pregnancies in the quiescent state, while disruption of autophagy balance (either increase or decrease) can promote labor. This evidence includes the following: a) phosphorylated mTOR (mammalian target of rapamycin), an autophagy inhibitor, is decreased (i.e. autophagy is increased) as pregnancy nears term in mice, rats and baboons^45^,^46^; b) in p53 conditionally deficient mice, a strain in which spontaneous preterm delivery occurs, the autophagy inducer rapamycin rescues the preterm delivery phenotype^47^; c) women with an unfavorable cervix who carry a polymorphism of the ATG16L1 gene resulting in decreased autophagy experienced shorter times of induction of labor than non-carriers^48^; d) autophagy is more pronounced in placentas obtained from women delivered by cesarean section than those delivered vaginally, possibly consistent with a role for withdrawal of autophagy in labor^49^; e) progesterone (the predominant hormone promoting uterine quiescence in pregnancy) induces autophagy in a mouse model of amyotrophic lateral sclerosis^50^.

a2V is a crucial molecule for the acidification of intracellular compartments and activation of lysosomal enzymes, coupled transport of small molecules and protein processing and degradation^25^. TLR stimulation is associated with downregulation of a2V^28^,^32^. Bafilomycin A1, an inhibitor of V-ATPase, disturbs intracellular pH and blocks autophagy by hampering the fusion of autophagosomes to lysosomes, preventing autophagosome turnover (34). Our results suggest that autophagic flux is altered in IPTL and with a2V blockade, through disturbances both in autophagosome formation (as reflected in a decrease in Atg proteins) and in autophagosome processing (as reflected in increased LC3BII protein despite diminished LC3B transcription, and a decrease in the lysosomal LAMP and a2V proteins). We show evidence supporting the hypothesis that this altered flux is mediated in part by the decrease of a2V enzyme, leading to decreased lysosomal function and a resultant accumulation of autophagosomes.

LAMP-1 and LAMP-2 deficiency in various metabolic conditions, neurodegenerative diseases and infectious diseases is linked with the accumulation of autophagosomes^42^,^51^,^52^,^53^. A decreased level of LAMP-2 was reported in LPS-induced experimental pancreatitis in rat^54^. Both LAMP-1 and LAMP-2 knockout mice are viable and fertile, however, an embryonic lethal phenotype occurs in LAMP-1 and LAMP-2 double knockout mice^42^. In the present study, LAMP-1 and LAMP-2 protein were diminished in preterm labor induced by both inflammatory and non-inflammatory stimuli. However, in non-inflammation induced preterm labor (NIPTL) a2V and LC3B paralogue expression remain unaffected. Together, these data suggest that the altered autophagy in IPTL may require both blockade of autolysosome formation (due to LAMP proteins deficiency) and decreased lysosomal function (through decreased activity of lysosomal enzyme a2V).

Autophagy also maintains cellular homeostasis by suppressing the activation of a master regulator of inflammatory pathways, NF-κB^43^ and other inflammatory mediators^55^. Our results suggest that alteration of autophagy in IPTL enhances the activation of NF-κB p65, thus helping to amplify inflammatory responses, in a process controlled in part by a2V. Our results are consistent with those of Chang et al, who showed that the lysosomal inhibitor Bafilomycin A1 leads to the accumulation of LC3B-II because of hindrance of lysosomal degradation, which subsequently increase NF-*κ*B p65 activation and secretion of proinflammatory cytokines^56^. Our study shows that specific blockade of a2V produces the same response as Bafilomycin A1, including perpetuation of the inflammatory response through persistent activation of NF-κB, suggesting that a2V and Bafilomycin A1 affect a common mediator.

Together with our previous work^32^ the present study shows how intrauterine infection can induce alteration of autophagy, suggesting a mechanism for the genesis of hyper-inflammatory processes in preterm labor. Our findings show that during inflammation-induced preterm labor the autophagic machinery becomes altered, leading to the accumulation of autophagosomes which could be linked with decreased levels of lysosomes and a2V in both uterus and placenta. This leads to enhancement of NF-κB signaling, amplification of inflammatory responses and subsequent labor (Fig. 8).

The significance of autophagy in various aspects of physiology is currently being recognized. Here for the first time, we show that disruption of this process is associated with inflammation-induced preterm labor. Better understanding of the molecular basis of autophagy and its regulation by and influence on regulators such as cytokines, chemokines, hormones, physical exercise and essential dietary management, may suggest methods to modify these pathways. Such modulation may lead to preventive or therapeutic interventions for preterm labor in the future.

## Methods

### Mice

All procedures involving animals were approved by the NorthShore University HealthSystem Animal Care and Use Committee and conform to the Guide for Care and Use of Laboratory Animals (1996, National Academy of Sciences). The methods were carried out in accordance with the approved guidelines. Mice used in the present studies were CD-1 strain. Female mice in estrus were selected by the gross appearance of the vaginal epithelium^57^ and were impregnated naturally. Mating was confirmed by the presence of a vaginal plug and the day of plug formation was counted as day 0.5 of pregnancy.

### Inflammation-induced preterm labor (IPTL) model

Labor was induced in mice on gestation day 14.5 as previously described^20^,^24^,^58^. Briefly, animals were anesthetized with 0.015 ml/g body weight of Avertin (2.5% tribromoethyl alcohol and 2.5% tert-amyl alcohol in PBS). A 1.5 cm midline incision was made in the lower abdomen. In the mouse, the uterus is a bicornuate structure in which the fetuses are arranged in a ‘beads-on-a-string' pattern. Intrauterine injections were performed in the midsection of the right uterine horn at a site between two adjacent fetuses, taking care not to inject individual fetal sacs. Mice underwent injection of (a) LPS (TLR4 agonist, extracted from *Salmonella enterica*, L2262, Sigma), 0.025 mg/mouse); or (b) the combination of PGN (TLR2 agonist, extracted from *Staphylococcus aureus,* 77140, Sigma), 0.3 mg/mouse) plus poly(I:C) (TLR3 agonist, 27-4729-01, Amersham Biosciences), 1.0 mg/mouse; or (c) PBS (control for both). PGN and poly(I:C) were combined because we showed previously that this combination produces dramatic synergy (both preterm delivery and inflammatory responses)^20^,^24^,^32^. Surgical procedures lasted approximately 10 minutes. The abdomen was closed in two layers, with 4-0 polyglactin sutures at the peritoneum and wound clips at the skin. Animals recovered in individual, clean cages in the animal facility. The doses of LPS and PGN+poly(I:C) used cause delivery within 18–24 h after injection.

### Non-inflammation induced preterm labor (NIPTL) model

Mifepristone (RU486, S-510, Enzo Life sciences), a progesterone receptor antagonist, was used to induce premature progesterone withdrawal^36^,^37^. Mifepristone was solubilized in DMSO and stored in −20°C. This stock was diluted in 1 M acetic acid and further diluted in water at the time of use. A dose of 150 μg/animal was injected subcutaneously on gestation day 14.5, predictably causing delivery within 18 h after injection. The same volume of DMSO in 1 M acetic acid in water was used as a vehicle control.

### Tissue harvest

Animals were euthanized 8–10 h after either surgery or injection. The inoculated/right horn was incised longitudinally along the anti-mesenteric border. Gestational tissues uteri (from regions inclusive of the decidual caps underlying placental attachment sites) and placentas were harvested, washed in ice-cold PBS, flash-frozen in liquid nitrogen and stored at −85°C for mRNA and protein extraction or fixed in 10% neutral buffered formalin for immunohistochemistry.

### Cell culture

The RAW 264.7 mouse macrophage cell line (American Type Culture Collection TIB-71) was cultured in DMEM High Glucose (11965-092, GIBCO) supplemented with 10% fetal bovine serum, 1% streptomycin and 1% penicillin in tissue culture flasks at 37°C in 5% CO_2_/95% air and were passaged every 2 or 3 days to maintain logarithmic growth. Prior to each experiment, cells (4 × 10^5^ cells per well) were plated in triplicate in 24-well plates and cultured overnight.

Cells were cultured for 30 min in the presence of PBS, PGN (1 μg/ml) plus poly(I:C) (10 μg/ml) or LPS (5 ng/ml), washed with PBS and then treated for 1 h, 2 h, 3 h or 5 h with either anti-a2V (5 μg/ml), IgG (same concentration as anti-a2V), bafilomycin A1 (a V-ATPase and autophagy inhibitor) (10 nM), DMSO (same volume as bafilomycin A1) or PBS. All *in-vitro* experiments were conducted in triplicate and repeated twice (i.e. three triplicate experiments). Viability of cultured cells was assessed using trypan blue dye exclusion. For RAW 264.7 cells, viability prior to plating was 95% and 3 h after plating was as follows for each treatment group: 93.5% for control (medium) treatment; 90% for LPS; 91.3% for PGN+poly(I:C); 90% for anti-a2V; 90.5% for anti-a2V+LPS; 88% for anti-a2V+PGN+poly(I:C). The differences between pre- and post-plating values were not statistically significant from each other.

### Decidual and placental cell preparation and *ex vivo* treatment

Uteri were dissected on day 14.5 of pregnancy and decidual caps and placentas were collected. Decidua and placenta were prepared as single-cell suspensions as described previously^59^. Briefly, tissues were minced in Hank's balanced salt solution (HBSS, Life technologies), mechanically dispersed through a 100-μm nylon filter, and centrifuged at 1500 rpm. The remaining pellet was dispersed in RPMI medium at 10^7^ cells/ml in 48-well plates. Prior to plating, placental suspensions underwent red cell lysis by incubation with red blood cell lysis buffer (BioLegend). The above specimens were incubated at 37°C in 5% CO_2_/95% air for 1 h. Viability of *ex vivo* cultured cells was >95% as assessed using the trypan blue dye exclusion test. Decidual and placental cells underwent treatment for 30 min in the presence of PBS, PGN (1 μg/ml) plus poly(I:C) (10 μg/ml) or LPS (5 ng/ml), and then treated for 2 or 5 h with either anti-a2V (5 μg/ml) or IgG (same concentration as anti-a2V). At the end of incubation medium was separated and cells were collected for protein extraction (see below). All *ex vivo* experiments were done thrice and in triplicate.

### Real-time PCR

Total RNA was extracted after homogenization in Trizol reagent. Quantity and integrity of RNA were confirmed by the ratio at 260:280 nm and electrophoresis was performed on a 1.5% native agarose gel to visualize 18 S and 28 S ribosomal RNA subunits. Samples were stored at −80°C until further use. Two μg of total RNA were used as a template for cDNA synthesis using qScript cDNA supermix (Quanta Biosciences).

Duplex RT-PCR was performed with one primer pair amplifying the gene of interest and the other an internal reference (GAPDH) in the same tube using the Applied Biosystems Step One Real-time PCR system. Thermocycler parameters were 50°C for 2 min, 95°C for 10 min, and then 40 cycles at 95°C for 15 sec and 60°C for 1 min. Semiquantitative analysis of gene expression was done using the comparative CT (ΔΔCT) method, normalizing expression of the gene of interest to that of GAPDH. PCR assays were performed in duplicate for each of the samples. The prevalidated Taqman gene expression assays for Atg4c (Mm01259886_m1), Atg7 (Mm00512209_m1), Atg3 (Mm00471287_m1), LC3B (Map1lc3B, Mm00782868_sH), a2V (Atp6v0a2, Mm00441848_m1), TNF (Mm00443258), IL-1β (Mm00434228) and internal control Gapdh (4352339E) were purchased from Applied Biosystems. Real-time PCR was performed using universal PCR master mix reagent (Applied Biosystems) in accordance with the manufacturer's manual. Reactions were performed in a 10 μL mixture containing 1 μL cDNA. PCR assays were performed in duplicate for each of the tissue samples.

### Antibodies

Primary antibodies were as follows: mouse anti-a2V (Covance, Denver, PA); rabbit anti-GAPDH (Cell signaling, Danvers, MA); rat anti-F4/80, rabbit anti-LC3B, rabbit anti-LAMP-1, rat anti-LAMP-2, rabbit anti-ATG3, rabbit anti-ATG4, rabbit anti-ATG7, rabbit anti-NF-κB p65 (all from Abcam). Secondary antibodies were as follows: goat anti-rabbit IgG-FITC, -mouse IgG AF-594, -rabbit IgG AF-594 (Invitrogen), rabbit anti-rat IgG-FITC (Abcam), goat anti-rabbit, -rat, -mouse IgG-HRP (Santa Cruz Biotechnology), donkey anti-rabbit IRDye-800CW (LI-COR Bioscience, Lincoln, NE) and EnVision + dual link System-Horseradish peroxidase (HRP) (Dako). Isotype control antibodies were as follows: control mouse IgG (R&D Systems); rat IgG isotype, mouse IgG isotype and rabbit IgG isotype (Abcam).

### Immunohistochemistry and immunofluorescence

The placenta and uterine horns were collected from control and treated groups. Tissues were fixed in 10% neutral-buffered formalin at 4°C overnight, rinsed in PBS and immersed in 30% sucrose solution at 4°C overnight or until tissue sank. Tissues were snap frozen in OCT (Tissue-Tek) in liquid nitrogen and stored at −80°C until further use. 5-μm sections from frozen tissues were mounted onto silane-coated glass slides (Dako) and stored at −80°C until used. The Dako EnVision + dual link System-HRP (DAB+) (Dako) was used to stain the frozen sections according to the manufacturer's instructions with slight modifications. The sections were submerged in sodium citrate buffer (pH = 6) and heated for 10 min in a microwave oven for antigen retrieval.

Sections were incubated with ATG4c, ATG7, ATG3, TNF (2 μg/ml) or NF-κB p65 (1 μg/ml) antibody overnight at 4°C. After washing, sections were incubated with secondary antibody EnVison+ dual link system-HRP labeled polymer anti-mouse and anti-rabbit IgG. The chromogen 3, 3'diaminobenzidine (DAB, brown) was used as substrate for the EnVision + dual link system HRP according to the manufacturer's instructions. The sections were counterstained with Mayer's hematoxylin and mounted in Faramount aqueous mounting medium (Dako). The immune-staining was evaluated by light photomicroscopy (Carl Zeiss) using a high resolution camera (Canon G10).

Sections were incubated with anti-F4/80 (1 μg/ml), anti-LC3B (1 μg/ml), anti-a2V (20 μg/ml), anti-LAMP-1 or anti-LAMP-2 (10 μg/ml) antibody overnight at 4°C, followed by incubation with the appropriate secondary antibody labeled with FITC or AF-594 for 45 min at room temperature. To visualize the nuclei cells were fixed in Prolong gold antifade reagent with DAPI (Invitrogen). After mounting the specimens on slides with Vectashield, antigen distribution was examined under a Nikon Eclipse TE2000-S florescence microscope (Nikon Instrument INC).

Mouse/rabbit/rat isotype control antibodies were used at the same concentrations as primary antibodies, and sections were incubated simultaneously with isotype control antibodies for all primary antibodies.

Tissue immunostaining results were scored negative if no immunopositive tissue was present. The total score was based on the percentage of stained tissue and immunostaining intensity. The percentage of stained tissue and immunostaining intensity was calculated according to the method described in Teixeira et al^60^. The immunostaining index score (ISIS) was generated by using the following equation: stained area score (SAS) multiplied by the immunostaining intensity score (IIS): (ISIS = SAS × IIS). The number of autophagosomes was counted manually (green dots were counted per 40X view). Where dots were difficult to distinguish from one another, they were counted as one, as described by Klionsky et al^61^.

### Immunocytochemistry

Immunocytochemistry was performed for LC3B, NF-κB p65, LAMP-2 and a2V in the RAW 264.7 mouse macrophage cell line. Briefly, RAW 264.7 cells were fixed with 4% paraformaldehyde, and permeabilized with 0.1% Triton X-100 and 3% BSA (Bovine Serum Albumin, Sigma-Aldrich). For the double immunostaining of LAMP-2 and a2V, cells were placed in 10 μg/ml and 20 μg/ml of antibody, respectively in 1% BSA-PBS overnight at 4°C. For the detection of LC3B and NF-κB p65, cells were incubated with 1 μg/ml of antibody overnight at 4°C. After washing with PBS-T, the specimens were incubated with the appropriate secondary antibody labeled with FITC (green) or AF-594 (red) for 45 min at room temperature. To visualize the nuclei, cells were counterstained with DAPI. After mounting the specimens on slides with Vectashield, antigen distribution was examined under a Nikon eclipse TE2000-S florescence microscope (Nikon Instrument INC). Experiments were repeated three times with duplicates. Mouse/rabbit isotype control antibodies were used at the same concentration as the primary antibodies.

### Protein extraction and Western blot analysis

For protein extraction cells were lysed and sonicated or tissues were homogenized in ice-cold 1X RIPA buffer (Santa Cruz Biotechnology) containing protease and phosphatase inhibitor (Roche Applied science). Lysates were incubated on ice for 30 min and centrifuged at 10,000 × g for 10 min at 4°C. Supernatant fluid was collected and used as a total cell lysates for protein assays. Samples were stored at −80°C until further use. Protein concentration was measured by BCA protein assay and spectrophotometry at A_280_ (Nanodrop 2000, Thermo Scientific). Equal amounts of protein (5–20 μg) from total cell lysates were separated by 4–12% SDS-PAGE and blotted onto PVDF transfer membranes. The membranes were blocked at room temperature for 1 h in 5% nonfat dry milk in TBS-T. Blots were incubated with primary antibody to ATG3, p62, LC3B, LAMP-1, LAMP-2 and/or GAPDH overnight at 4°C and with appropriate secondary antibody for 1 h at room temperature. Quantification of bands was performed using ImageQuant software (Molecular Dynamics). Background intensity was subtracted from each sample and then fold change was determined. Fluorescent blots were imaged on the Odyssey Infrared Imaging System (LI-COR Biosciences). To verify equal loading, membranes were either cut in half and probed for target and loading controls simultaneously or stripped and re-probed for GAPDH as appropriate.

### Cytokine/chemokine bioassay

The secretion of a panel of mouse cytokines/chemokines was analyzed by Milliplex map kit (Millipore) in the supernatant recovered from the RAW 264.7 mouse macrophage cell line and assayed on a MAGPIX instrument (Millipore) as per the instructions provided by manufacture. Equal amounts of cell culture supernatant were used and assay was repeated three times with duplicates.

### NF-κB p65 concentration measurement

Concentration of total and phospho-NF-κB p65 was measured using InstantOne ELISA Kit (eBioscience) in total cell lysate from uterus, placenta and RAW 264.7 cells^62^ and assayed on a fluorometer (Bio-Tek Instruments) as per the instruction provided by manufacture. Equal amounts of protein (50 μg) were used for the assay. Concentration was measured in relative fluorescence unit (RFU)/μg of protein. The assay was run in duplicates with n = 4 in each group.

### Statistical analysis

Continuous variables (e.g. relative mRNA levels) were assessed with Student's t-test or ANOVA or, when data were not normally distributed and two groups were compared, the Mann-Whitney U test.

## Author Contributions

V.A. and M.K.J. designed and performed research, analyzed data, and wrote the paper. T.M. and G.K.K. performed research. A.G.S. analyzed data, and helped in writing the paper. K.D.B. and E.H. designed research, analyzed data, and helped in writing the paper.

## Supplementary Material

Supplementary Information Supplementary Information
