# Supplementary material for: Altered autophagic flux enhances inflammatory responses during inflammation-induced preterm labor
Source: Sci Rep. 2015 Mar 23;5:9410. doi: 10.1038/srep09410 (PMC4369745; doi:10.1038/srep09410)
Supplement: Supplementary Information [file srep09410-s1.doc]

**Supplementary information for the manuscript titled**

**Altered autophagic flux enhances inflammatory responses during inflammation-induced preterm labor**

**Varkha Agrawal^#,§^, Mukesh K. Jaiswal^$,§^, Timothy Mallers^$^, Gajendra Katara^$^, Alice Gilman-Sachs^$^, Kenneth D. Beaman^$^ and Emmet Hirsch^#*,¶^**


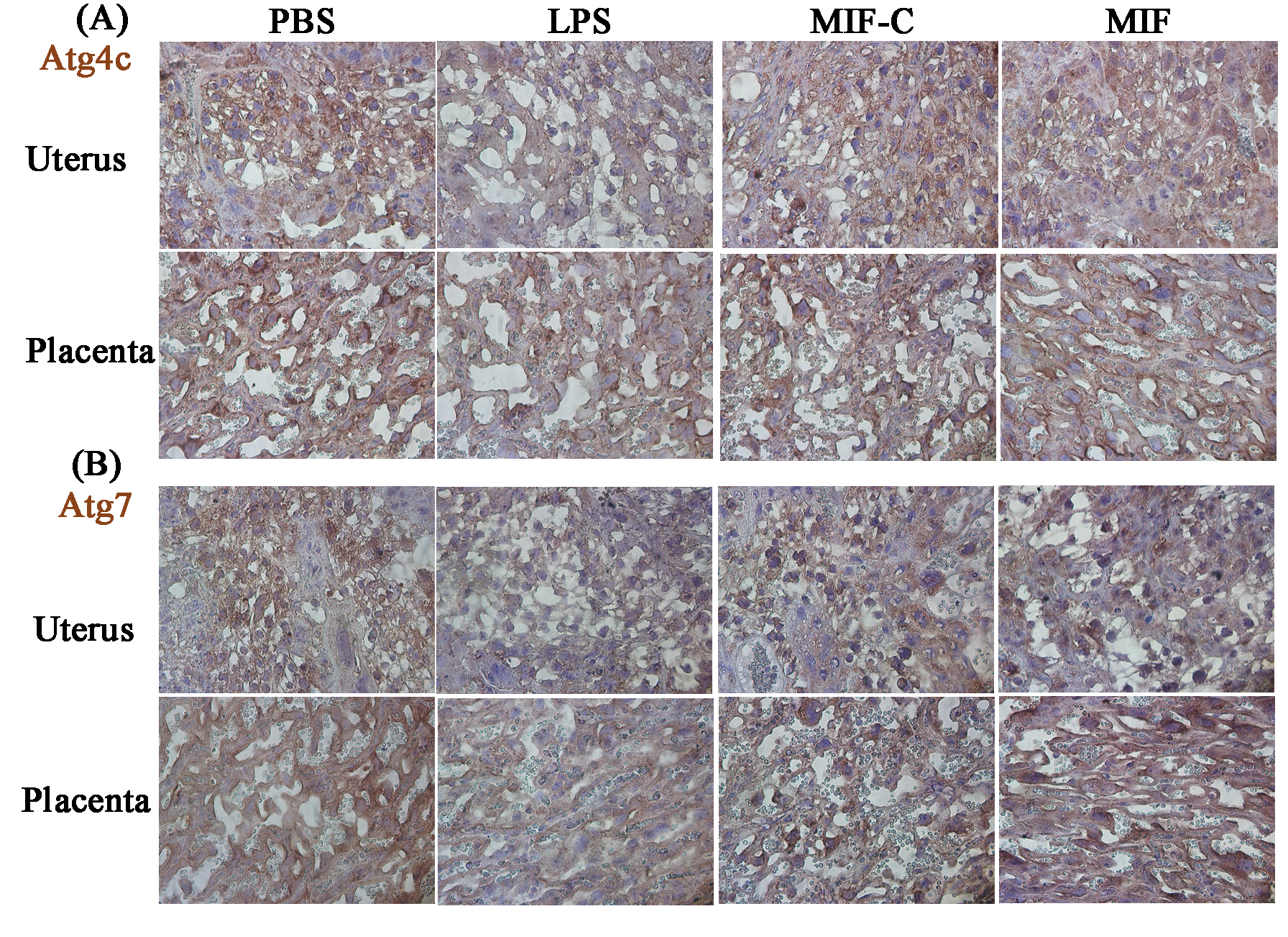


**Supplementary Figure 1: Expression of Atg4c and Atg7 is decreased in inflammation-induced preterm labor.** Distribution of Atg4c (A) and Atg7 (B) (brown) in uterus and placenta. N=4-5 each group. Six sections per animal were analyzed. Original magnification: 400X. PBS: intrauterine PBS on day 14.5; LPS: intrauterine injections on day 14.5, MIF-C: subcutaneous DMSO control on day 14.5; MIF: subcutaneous mifepristone in DMSO on day 14.5.


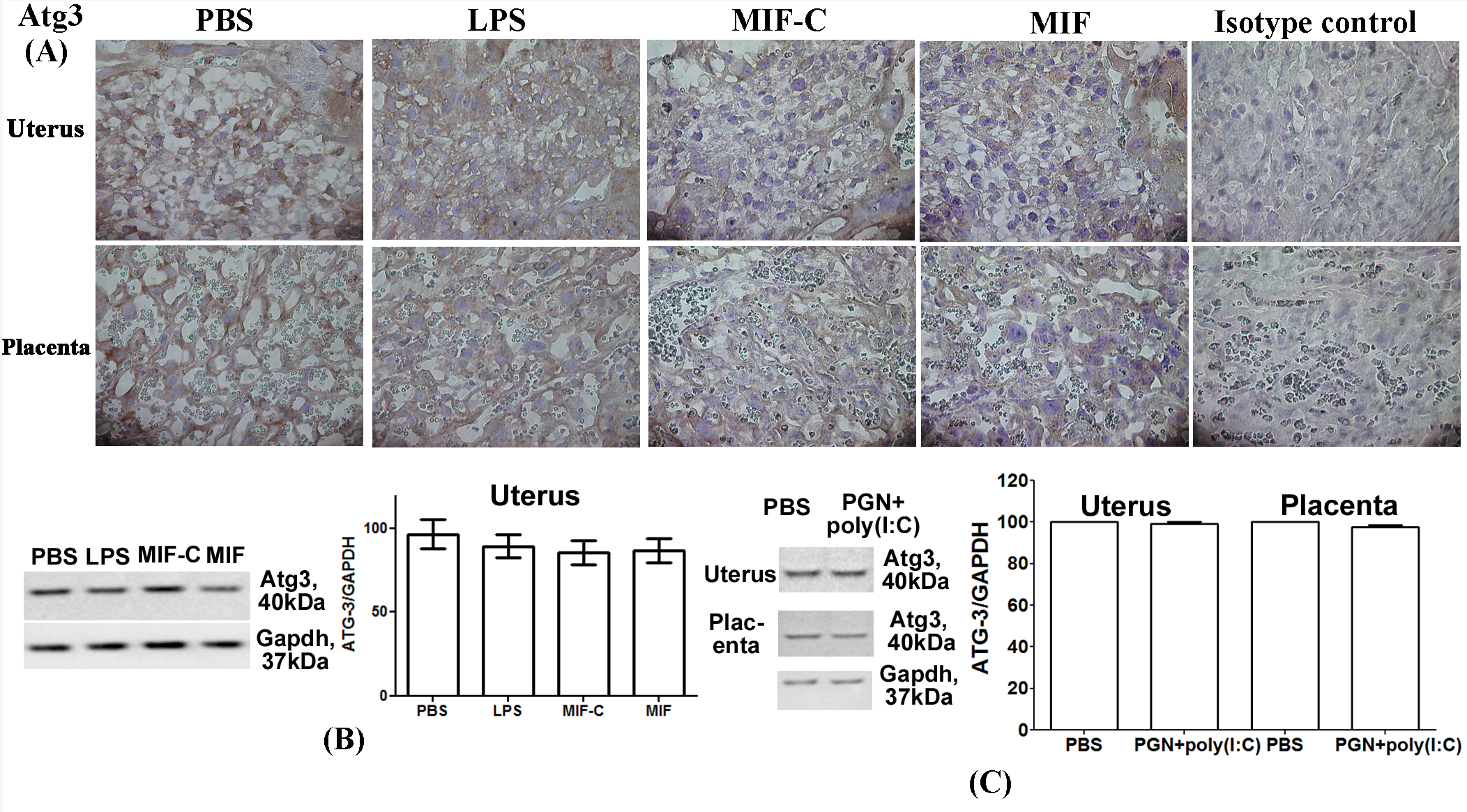


**Supplementary Figure 2: Expression of Atg3 in inflammation-induced preterm labor.** Distribution of Atg3 (brown) in uterus and placenta (A). N=4-5 each group. Six sections per animal were analyzed. Original magnification: 400X. Panels B and C show western blots of Atg3 and GAPDH in uterus and placenta with corresponding densitometric analysis (N=4-5 each group). PBS, LPS and PGN+poly(I:C): intrauterine injections on day 14.5; MIF-C: subcutaneous DMSO control, MIF: subcutaneous mifepristone in DMSO on day 14.5. Error bars=±SEM. *P≤0.05, **P≤0.01 Significant difference vs. respective control.


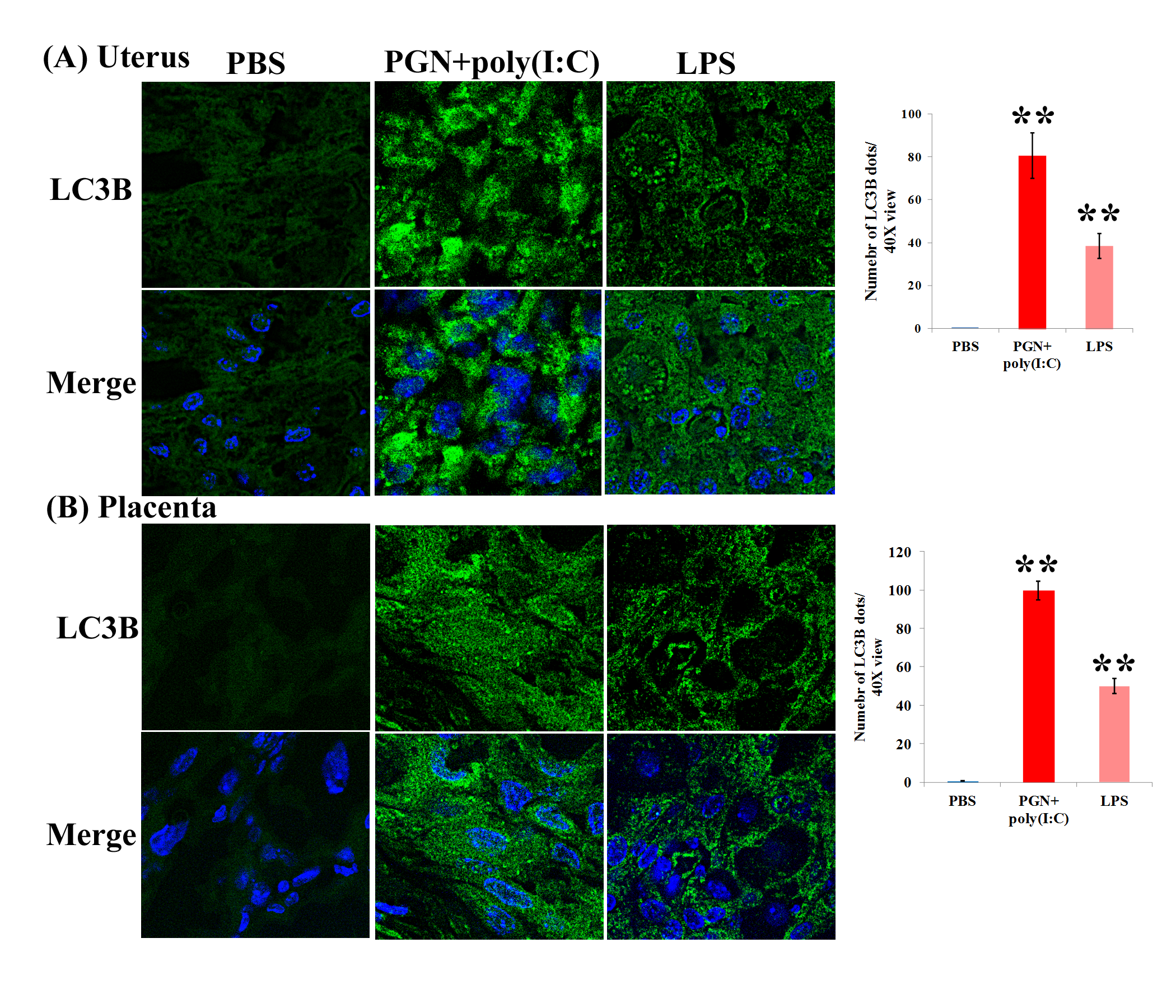
**Supplementary Figure 3: Accumulation of LC3B puncta in decidual and placental tissues in inflammation induced preterm labor.** Uterus shown in (A) and placenta in (B) with adjacent graph showing counting of LC3B puncta at 400X. LC3B stained in green and nuclei stained with DAPI (blue) in merged images. N=4-5 each group. Six sections per animal were analyzed. Original magnification: 400X. PBS, LPS and PGN+poly(I:C): intrauterine injections on day 14.5.

**Supplementary Figure 4: Expression of LC3B is increased in decidual macrophages in inflammation induced preterm labor.** Macrophages stained with F4/80 (green), autophagosomes with LC3B (red), and merged images of F4/80 and LC3B in uterus recovered from preterm labor and control groups. Isotype controls are also shown. N=4-5 each group. Six sections per animal were analyzed. Original magnification: 400X. PBS, LPS and PGN+poly(I:C): intrauterine injections on day 14.5.


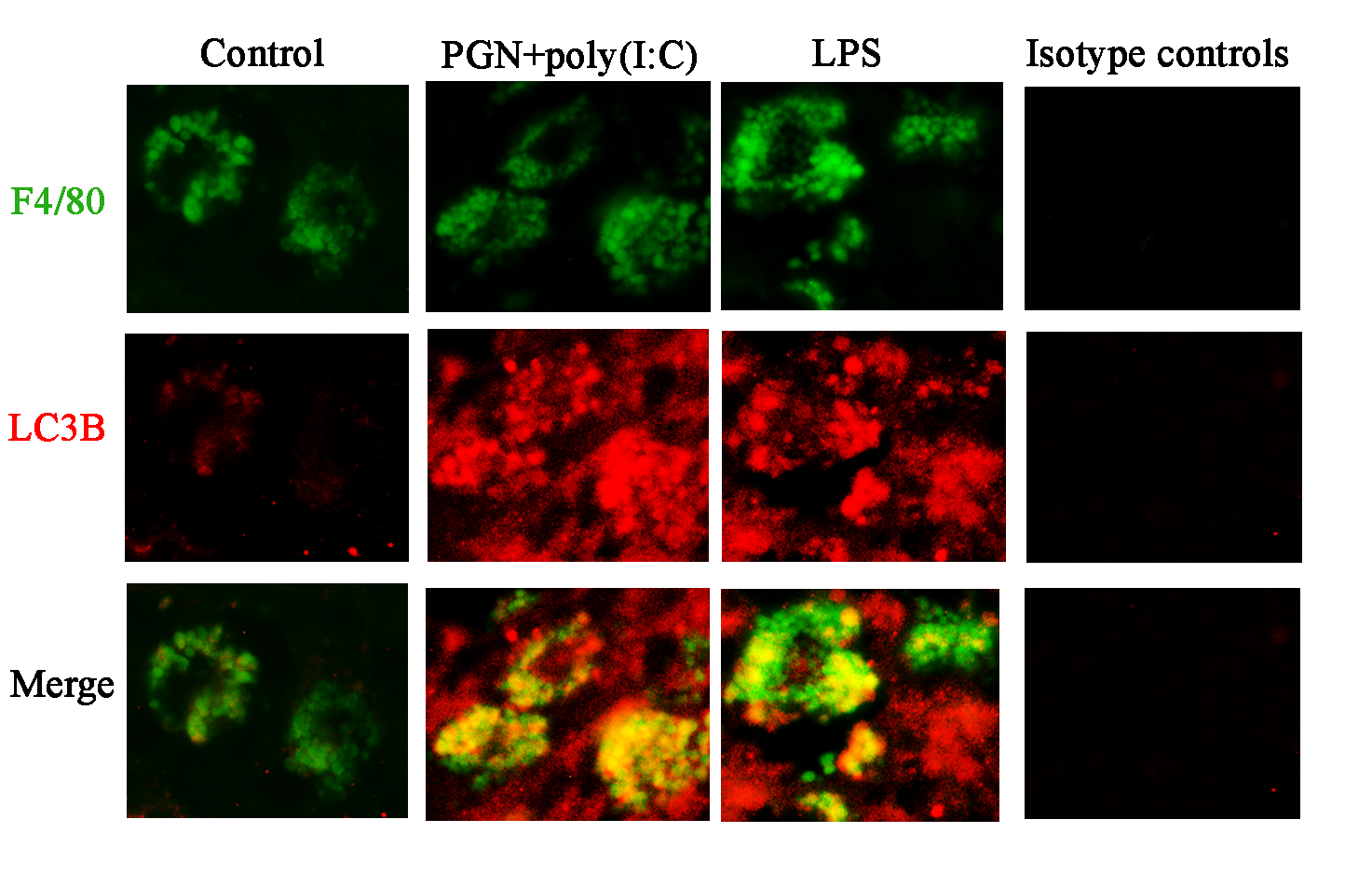


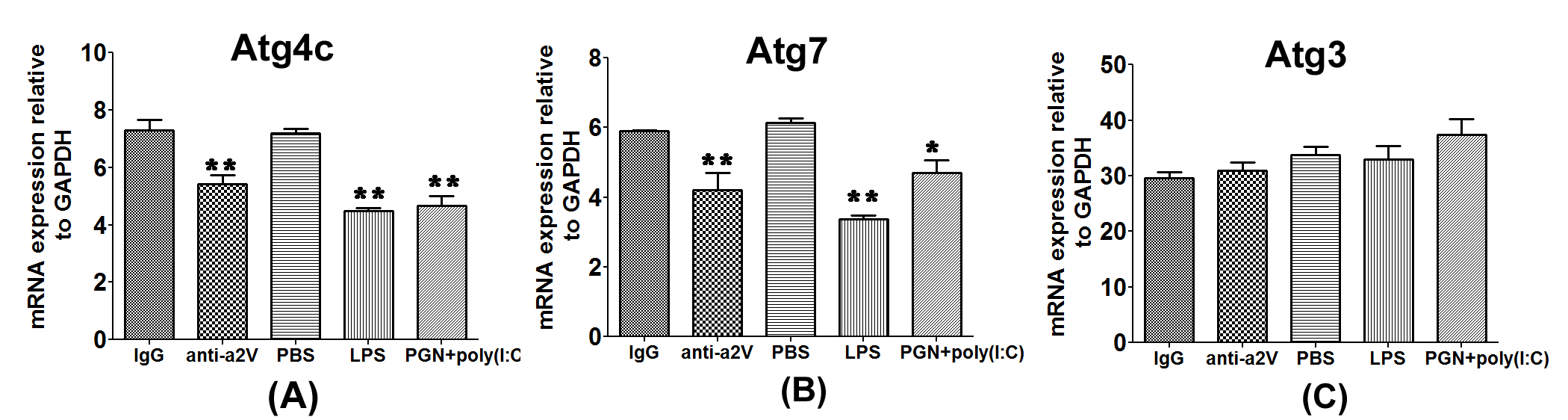


**Supplementary Figure 5: Expression of Atg4c, Atg7 and Atg3 in RAW 264.7 cells.** Panels A-C show mRNA expression of Atg4c, Atg7 and Atg3 in RAW 264.7 cells treated with IgG control, anti-a2V antibody, PBS, LPS or PGN+poly(I:C). Each experiment was done three times with triplicates. Error bars=±SEM. *P≤0.05, **P≤0.01 Significant difference vs. respective control.


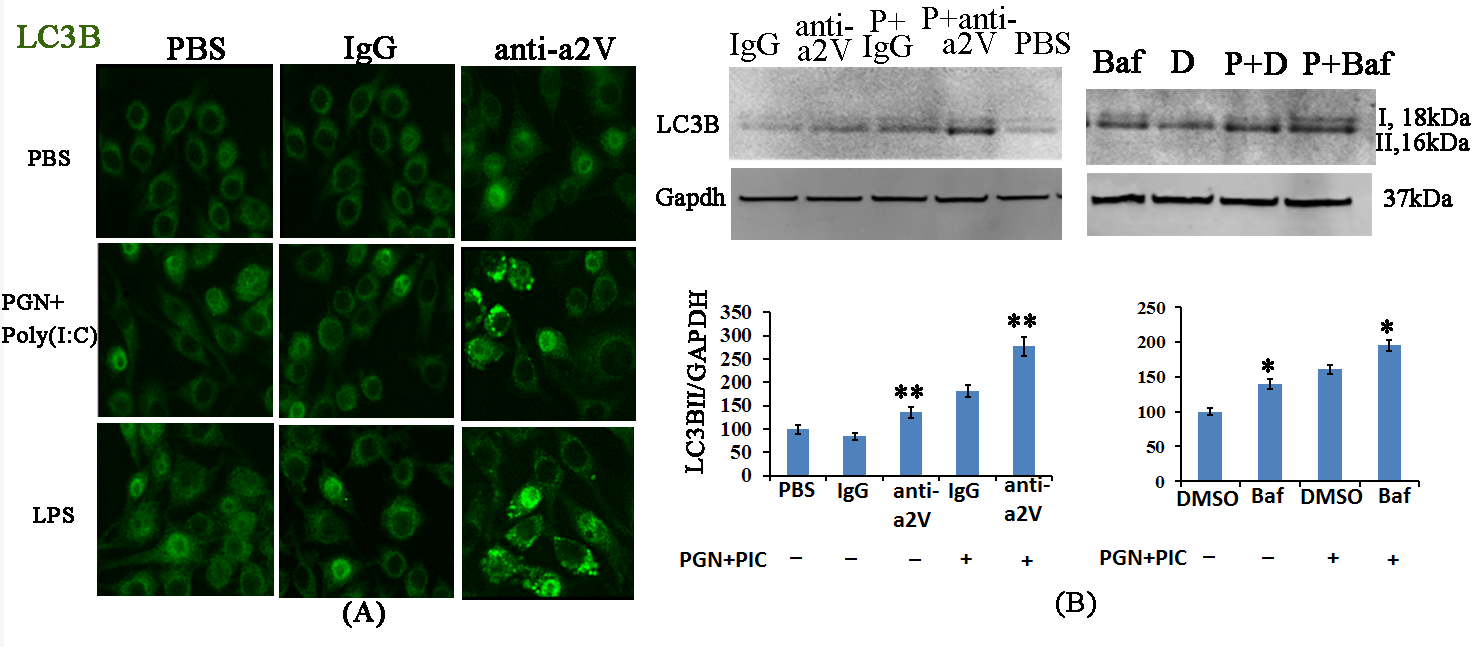
**Supplementary Figure 6: Blocking of a2V increases LC3B in the RAW 264.7 mouse macrophage cell line.** Panel A shows expression of LC3B (green) 30 min after incubation with PBS (control), PGN+poly(I:C) or LPS, followed by 90 min incubation with PBS, IgG control or anti-a2V. Original magnification: 400X. Panel B shows western blots of LC3B-I, LC3B-II and GAPDH and corresponding densitometry of LC3B-II and GAPDH. P=PGN+poly(I:C); Baf= Bafilomycin A1; D=DMSO. Each experiment was performed three times in triplicate. Original magnification: 400X. Error bars=±SEM. *P≤0.05, **P≤0.01 Significant difference vs. respective control.

**Supplementary Figure 7: Isotype controls for RAW 264.7 cells.** FITC (green), AF-594 (red) and merged together with the nuclear stain DAPI (blue). Original magnification: 200X.


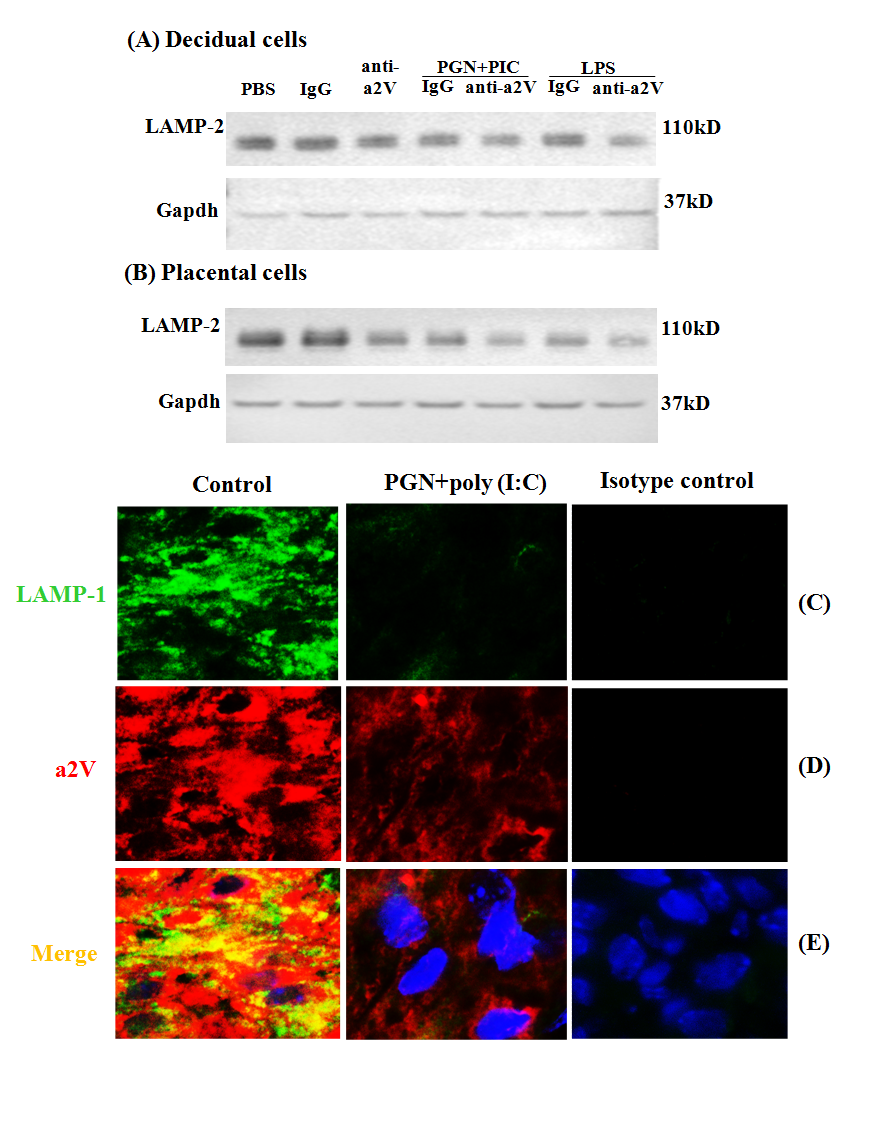


**Supplementary Figure 8: Decrease of a2V is associated with reduction of LAMP-2 in the decidual and placental cells.** Decidual (A) and placental (B) cells were treated with PBS, PGN+poly(I:C) or LPS in the absence or presence of an a2V neutralizing antibody and western blots of LAMP-2 and GAPDH were preformed. Each experiment was performed three times in triplicate. **a2V and LAMP-1 co-localize and are suppressed with IPTL.** LAMP-1 labeled in green (C); a2V labeled in red (D) and merged images (E) are shown together with the nuclear stain DAPI (blue) in uterus from control and PGN+poly(I:C)-treated animals. Panels show isotype controls. N=4-5 each group. Six sections per animal were analyzed. PBS and PGN+poly(I:C): intrauterine injections on day 14.5. Original magnification: 400X.

**
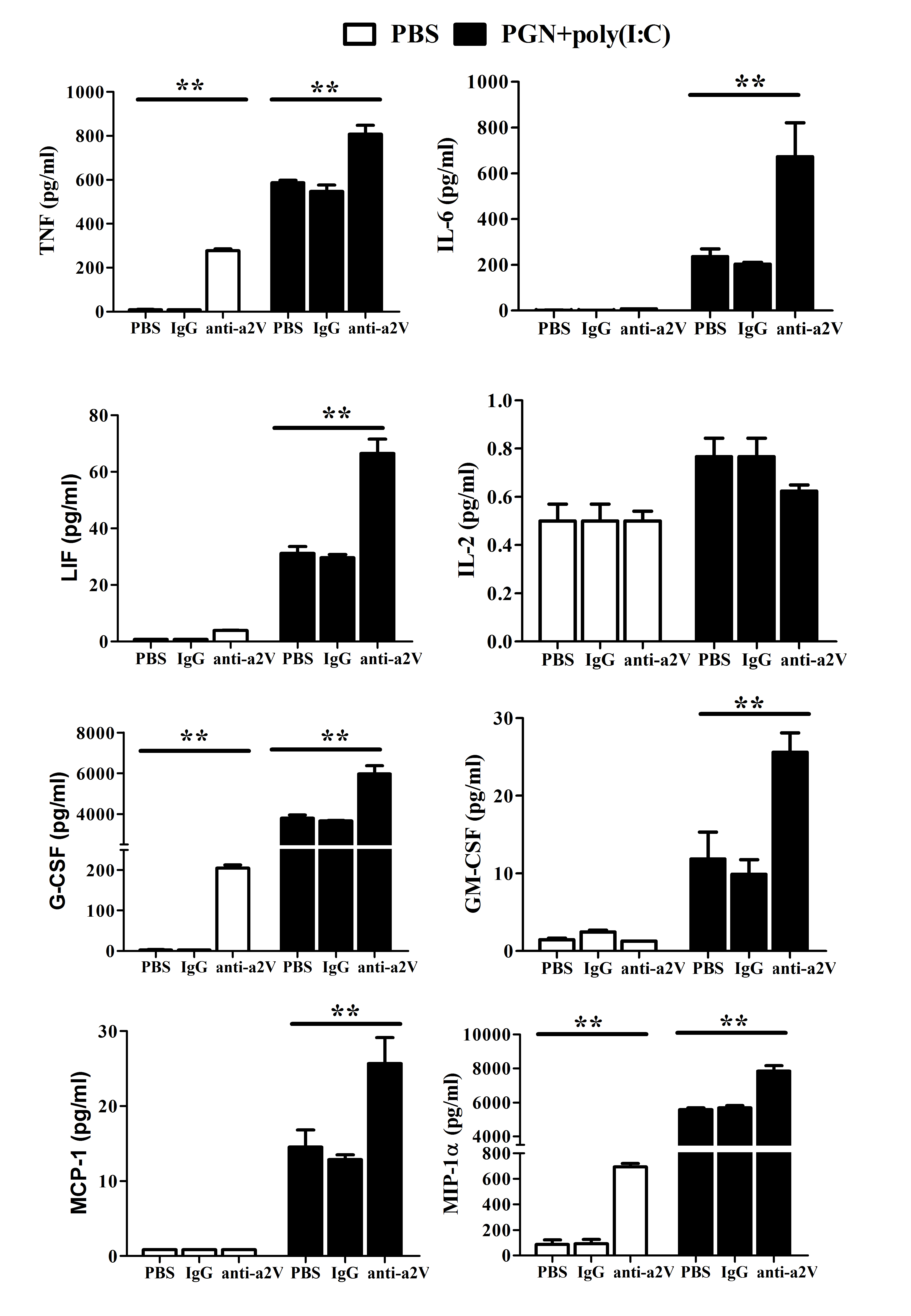
**

**Supplementary Figure 9: Blockade of a2V is linked with inflammation in RAW cells.** Pro-inflammatory cytokines and chemokines were measured by Luminex assay in medium from RAW 264.7 cells treated with PBS (control) and PGN+poly(I:C) for 30 min, followed by treatment with either PBS, IgG control or anti-a2V up to 5h. N=6 each group. Error bars=±SEM. **P≤0.01 Significant difference between PBS or PGN+poly(I:C) treated with PBS/IgG vs. treated with anti-a2V.


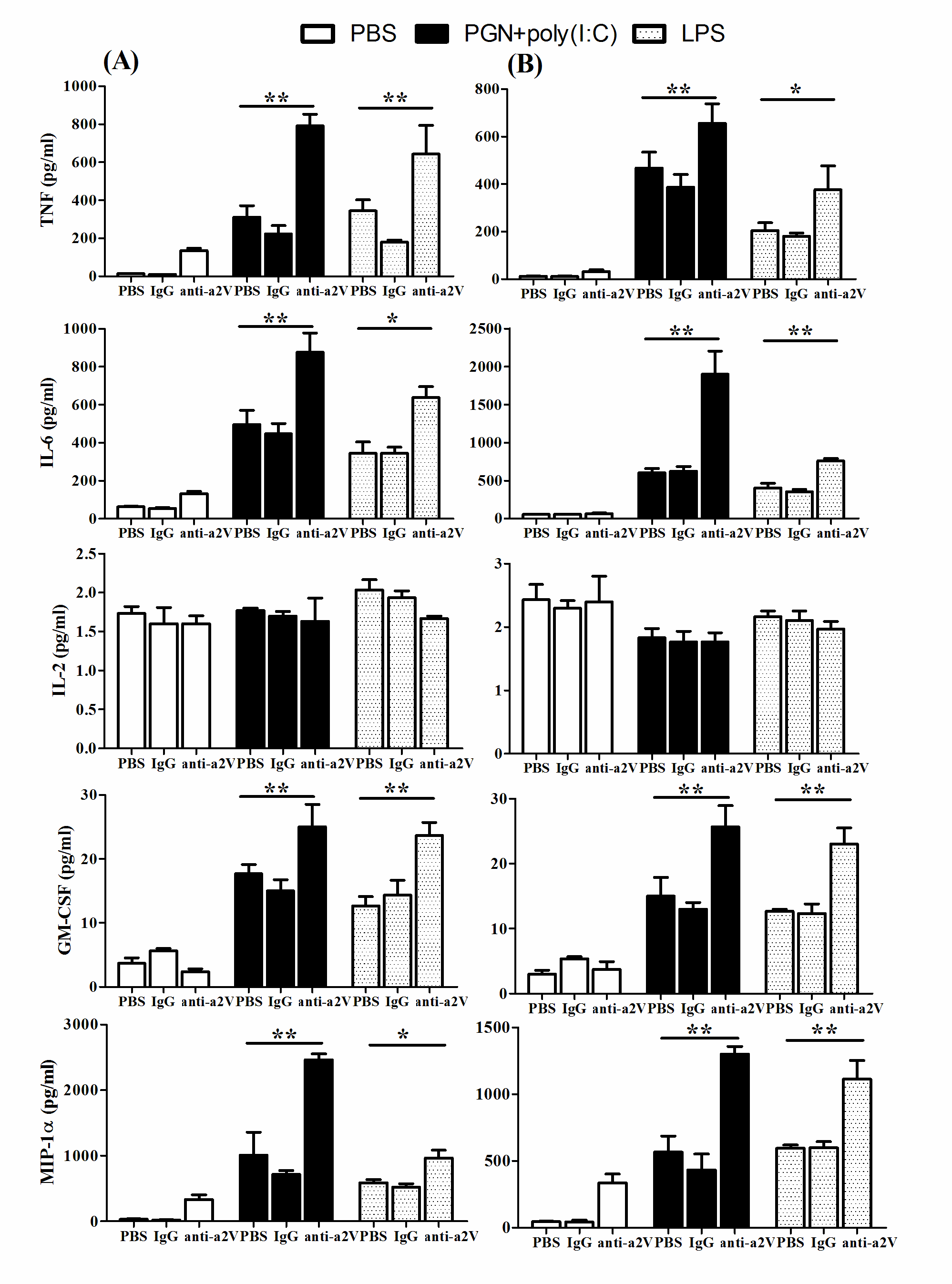
**Supplementary Figure 10: Blockade of a2V is linked with inflammation in decidual and placental cells.** Pro-inflammatory cytokines and chemokines were measured in medium from decidual (A) and placental (B) cells as treated, measured and described in Supplementary Figure 9. N=6 each group. Error bars=±SEM. *P≤0.05 , **P≤0.01 Significant difference between PBS and PGN+poly(I:C) or LPS treated with PBS/IgG vs. treated with anti-a2V.

**Supplementary Figure 11: Anti-a2V enhances LPS-induced mRNA expression of cytokines in the RAW 264.7 macrophage cell line.** Expression of TNF and IL-1β by RT-PCR in RAW 264.7 cells 30 min after incubation with PBS or LPS (5ng/ml), followed by 90 min incubation with anti-a2V (5μg/ml) or IgG (5μg/ml). n=3 replicates per condition per experiment. Depicted is a representative figure from three repeat experiments. P values were calculated by one way ANOVA and compare exposures with and without anti-a2V. Error bars=±SEM. ^a^P≤0.01 significant difference LPS + IgG vs LPS+anti-a2V and ^b^P≤0.01 significant difference anti-a2V vs. IgG.


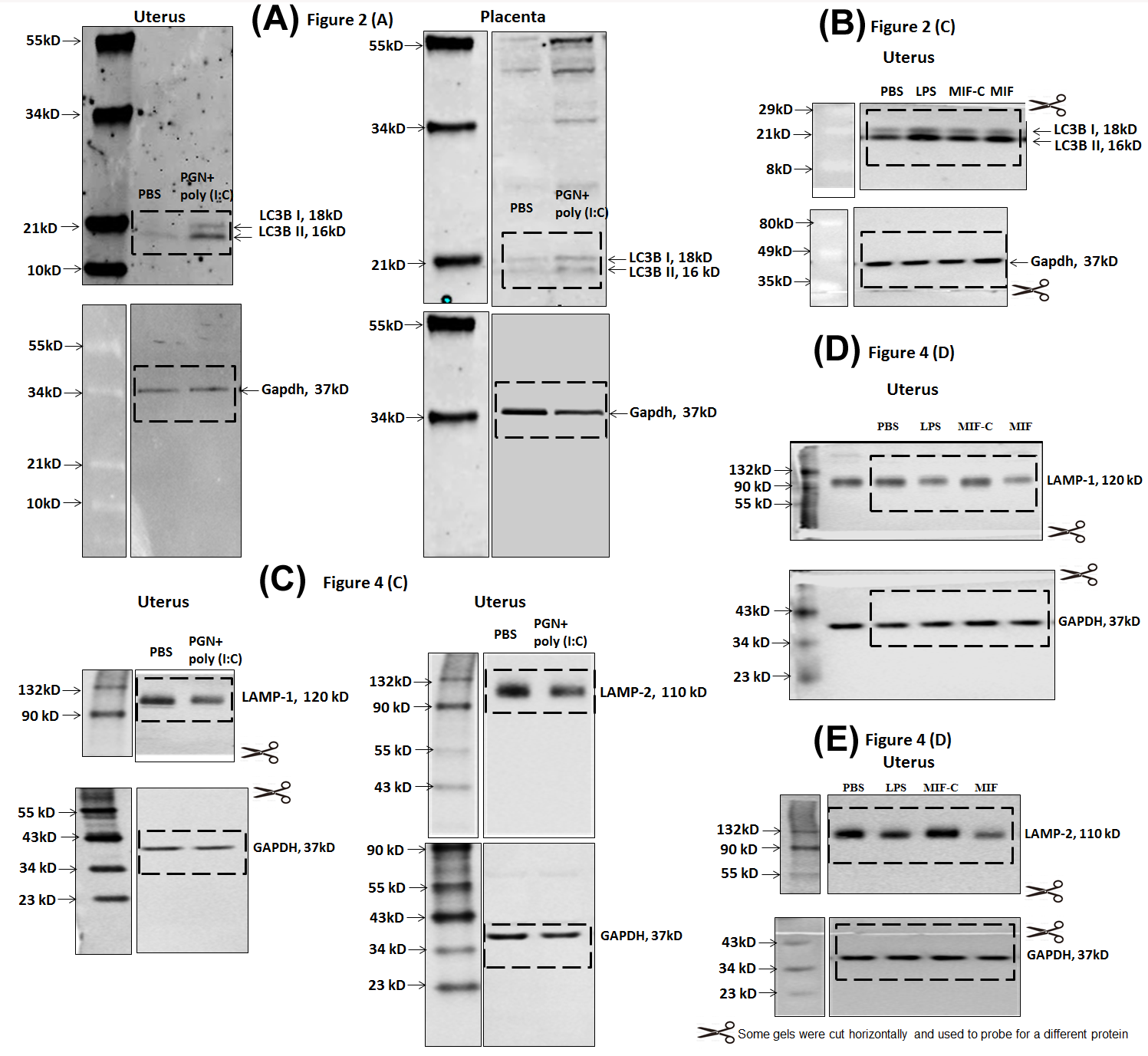
**Supplementary Figure 12: Full-length blots for figures 2 and 4**. LC3B-I and –II (A) For figure 2A, (B) for figure 2C; LAMP-1 and LAMP-2 (C) For figure 4C, (D) and (E) for figure 4D. Some gels were cut horizontally to probe for a different protein (or GAPDH).
